# Supplementary material for: Associations between compensable injury, perceived fault and pain and disability 1 year after injury: a registry-based Australian cohort study
Source: BMJ Open. 2017 Oct 5;7(10):e017350. doi: 10.1136/bmjopen-2017-017350 (PMC5639991; doi:10.1136/bmjopen-2017-017350)
Supplement: Supplementary file 1 [file bmjopen-2017-017350supp001.pdf]

**Supplementary Table 1**

*Multiple regression for association between injury characteristics and pain interference (only for pain intensity>0; N=370), EQ-5D, and GOS-E recovery outcome.*

| | | **Pain Interference** | | | | **EQ-5D Summary Score** | | | | **GOS-E Functional Outcome** | | | |
|---|---|---|---|---|---|---|---|---|---|---|---|---|---|
| **Injury characteristics** | | **M** | **(sd)** | **β** | **(95% CI)** | **M** | **(sd)** | **β** | **(95% CI)** | **N** | **%** | **OR** | **(95% CI)** |
| **Comorbidities** | None | 2.91 | 2.42 | Ref | | 0.82 | 0.22 | Ref | | 137 | 50.6 | Ref | |
| | Present | 3.27 | 2.62 | 0.23 | -0.0.32,0.78 | 0.77 | 0.25 | -0.05 | -0.10,0 | 73 | 47.1 | 0.86 | 0.56,1.31 |
| **Work status at injury** | Not working | 3.97 | 2.87 | Ref | | 0.72 | 0.33 | Ref | | 37 | 68.5 | Ref | |
| | Working | 2.91 | 2.41 | **-1.00** | **-1.87,-0.13** | 0.82 | 0.21 | **0.10** | **0.01,0.19** | 173 | 46.5 | **0.40** | **0.20,0.80** |
| **Injury place** | At home | 2.48 | 2.39 | Ref | | 0.82 | 0.24 | Ref | | 44 | 57.9 | Ref | |
| | Traffic/Road | 3.60 | 2.61 | **1.02** | **0.26, 1.78** | 0.77 | 0.25 | -0.03 | -0.10,0.04 | 66 | 39.5 | 0.67 | 0.37,1.23 |
| | Workplace | 3.60 | 2.38 | **1.21** | **0.26, 2.16** | 0.74 | 0.25 | -0.08 | -0.17,0.01 | 14 | 31.1 | **0.37** | **0.17,0.80** |
| | Other | 2.43 | 2.24 | 0.15 | -0.62, 0.92 | 0.86 | 0.18 | 0.04 | -0.03,0.10 | 86 | 62.3 | 1.37 | 0.74,2.26 |
| **Injury severity** | AIS region count | | | **0.37** | **0.14, 0.61** | | | **-0.03** | **-0.06,-0.01** | | | **0.70** | **0.58,0.82** |
| **Hospital stay** | 1-2 days | 2.48 | 2.35 | Ref | | 0.83 | 0.24 | Ref | | 95 | 60.5 | Ref | |
| | 3-6 days | 3.26 | 2.46 | **0.72** | **0.06, 1.37** | 0.81 | 0.22 | -0.01 | -0.06,0.05 | 69 | 52.3 | 0.80 | 0.49,1.30 |
| | 7-13 days | 2.88 | 2.52 | 0.30 | -0.49, 1.09 | 0.82 | 0.19 | 0.01 | -0.05,0.07 | 35 | 43.8 | 0.61 | 0.34,1.10 |
| | ≥ 14 days | 4.34 | 2.49 | **1.65** | **0.76, 2.55** | 0.70 | 0.27 | **-0.10** | **-0.19,-0.02** | 10 | 17.9 | **0.18** | **0.08,0.42** |
| **Type of discharge** | Home | 2.65 | 2.29 | Ref | | 0.84 | 0.22 | Ref | | 168 | 55.6 | Ref | |
| | Rehabilitation | 3.91 | 2.72 | **0.99** | **0.38, 1.61** | 0.73 | 0.25 | **-0.08** | **-0.14,-0.03** | 42 | 33.9 | **0.51** | **0.32,0.82** |

Supplementary tables

| | | | | | | | | | | | | | |
|---|---|---|---|---|---|---|---|---|---|---|---|---|---|
| **Compensation status** | None | 2.53 | 2.32 | Ref | | 0.84 | 0.22 | Ref | | 155 | 58.7 | Ref | |
| | TAC/Worksafe | 3.82 | 2.57 | **1.16** | **0.61, 1.71** | 0.75 | 0.25 | **-0.07** | **-0.12,-0.02** | 55 | 34 | **0.44** | **0.28,0.67** |
| **Fault** | At fault | 2.42 | 2.14 | Ref | | 0.85 | 0.19 | Ref | | 124 | 57.9 | Ref | |
| | Not at fault | 3.63 | 2.68 | **1.15** | **0.66, 1.64** | 0.76 | 0.26 | **-0.09** | **-0.13,-0.05** | 83 | 39.9 | **0.51** | **0.34,0.76** |

*Notes*: all analysis adjusted for age, sex and injury severity, except for the injury severity analysis which only adjusted for age and sex.

Supplementary tables

Supplementary Table 2

*Multiple regression for association between injury characteristics, pain catastrophising, pain self-efficacy, kinesiophobia and injustice experience.*

| | | PCS | | | | PSEQ | | | |
|---|---|---|---|---|---|---|---|---|---|
| **Injury characteristics** | | **M** | **(sd)** | **β**** | **(95% CI)** | **M** | **(sd)** | **β**** | **(95% CI)** |
| **Comorbidities** | None | 9.54 | 11.23 | Ref | | 45.63 | 14.19 | Ref | |
| | Present | 9.30 | 10.6 | 0.32 | -1.31,1.94 | 45.02 | 14.68 | -0.34 | -2.68,2.01 |
| **Work status at injury** | Not working | 11.65 | 12.61 | Ref | | 40.99 | 15.12 | Ref | |
| | Working | 9.12 | 10.71 | -0.06 | -2.57,2.44 | 46.63 | 14.46 | 1.71 | -1.22,4.65 |
| **Injury place** | At home | 8.18 | 10.67 | Ref | | 47.41 | 12.01 | Ref | |
| | Traffic/Road | 11.38 | 11.81 | 0.85 | -1.49,3.20 | 42.7 | 15.27 | -2.32 | -5.09,0.46 |
| | Workplace | 11.13 | 11.3 | 0.52 | -2.48,3.53 | 43.11 | 15.41 | -1.44 | -6.26,3.38 |
| | Other | 7.18 | 9.49 | -1.27 | -3.55,1.00 | 48.43 | 13.37 | -0.10 | -2.97,2.76 |
| **Injury severity** | AIS region count | | | 0.28 | -0.51,1.06 | | | -0.94 | -1.96,0.08 |
| **Hospital stay** | 1-2 days | 7.91 | 10.00 | Ref | | 49.11 | 12.57 | Ref | |
| | 3-6 days | 9.77 | 11.3 | 0.69 | -1.31,2.68 | 43.81 | 14.99 | **-3.34** | **-6.05,-0.63** |
| | 7-13 days | 9.24 | 10.55 | 1.94 | -0.09,3.98 | 45.33 | 13.96 | **-4.02** | **-7.27,-0.77** |
| | ≥ 14 days | 13.25 | 12.76 | 2.40 | -0.31,5.11 | 38.89 | 15.5 | **-5.70** | **-9.16,-2.23** |
| **Type of discharge** | Home | 7.92 | 9.67 | Ref | | 47.41 | 13.61 | Ref | |
| | Rehabilitation | 13.08 | 12.97 | **2.56** | **0.63,4.50** | 40.71 | 15.01 | **-2.76** | **-5.10,-0.42** |
| **Compensation status** | None | 8.04 | 10.12 | Ref | | 47.91 | 13.29 | Ref | |
| | TAC/Worksafe | 11.67 | 11.93 | 1.06 | -0.72,2.84 | 41.43 | 15.12 | **-3.32** | **-5.55,-1.10** |

Supplementary tables

| | | | | | | | | | |
|---|---|---|---|---|---|---|---|---|---|
| **Fault** | At fault | 7.22 | 9.72 | Ref | | 48.94 | 12.07 | Ref | |
| | Not at fault | 11.67 | 11.80 | **2.11** | **0.57,3.65** | 41.87 | 15.63 | **-4.29** | **-6.45,-2.11** |

*Notes*: all analysis adjusted for age, sex and injury severity, except for the injury severity analysis which only adjusted for age and sex.

Supplementary tables

(Supplementary Table 2 continued)

| Injury characteristics | | TSK | | | | IEQ | | | |
|---|---|---|---|---|---|---|---|---|---|
| | | **M** | **(sd)** | **β**** | **(95% CI)** | **M** | **(sd)** | **β**** | **(95% CI)** |
| **Comorbidities** | None | 36.99 | 8.32 | Ref | | 15.99 | 13.6 | Ref | |
| | Present | 37.32 | 7.99 | 0.80 | -0.65,2.24 | 16.67 | 13.75 | 1.14 | -1.17,3.46 |
| **Work status at injury** | Not working | 37.81 | 9.33 | Ref | | 17.68 | 14.27 | Ref | |
| | Working | 37 | 8.01 | 0.47 | -1.70,2.64 | 16.02 | 13.55 | 0.80 | -2.83,4.42 |
| **Injury place** | At home | 36.97 | 7.86 | Ref | | 12.57 | 11.06 | Ref | |
| | Traffic/Road | 37.53 | 8.05 | -0.93 | -2.82,0.95 | 19.22 | 14.43 | **4.20** | **1.25,7.16** |
| | Workplace | 39.76 | 7.63 | 0.96 | -1.55,3.48 | 19.39 | 13.6 | **4.20** | **0.08,8.32** |
| | Other | 35.79 | 8.56 | -1.78 | -3.85,0.29 | 13.51 | 12.97 | 1.03 | -2.17,4.24 |
| **Injury severity** | AIS region count | | | -0.20 | -0.76,0.35 | | | 0.95 | -0.02,1.91 |
| **Hospital stay** | 1-2 days | 36.66 | 7.77 | Ref | | 12.54 | 12.57 | Ref | |
| | 3-6 days | 37.45 | 8.62 | -0.14 | -1.82,1.54 | 17.41 | 13.82 | **3.71** | **1.04,6.37** |
| | 7-13 days | 36.98 | 8.1 | 0.44 | -1.47,2.36 | 16.81 | 13.11 | **4.84** | **1.50,8.19** |
| | ≥ 14 days | 37.77 | 8.64 | -0.96 | -3.41,1.49 | 22.55 | 14.14 | **7.05** | **3.26,10.34** |
| **Type of discharge** | Home | 36.52 | 8.14 | Ref | | 14.09 | 12.87 | Ref | |
| | Rehabilitation | 38.53 | 8.18 | 0.74 | -0.85,2.33 | 21.31 | 14.12 | **4.35** | **1.88,6.81** |
| **Compensation status** | None | 36.3 | 8.05 | Ref | | 13.66 | 12.51 | Ref | |
| | TAC/Worksafe | 38.37 | 8.28 | 0.86 | -0.58,2.31 | 20.31 | 14.39 | **3.95** | **1.60,6.31** |

Supplementary tables

| | | | | | | | | | |
|---|---|---|---|---|---|---|---|---|---|
| **Fault** | At fault | 36.19 | 7.79 | Ref | | 11.94 | 11.1 | Ref | |
| | Not at fault | 38.03 | 8.54 | 0.54 | -0.81,1.89 | 20.5 | 14.62 | **6.35** | **4.23,8.48** |

*Notes*: all analysis adjusted for age, sex, pain intensity and injury severity.

Supplementary tables

Supplementary Table 3a

*Direct and indirect effects between predictors and pain interference, adjusting for age, sex, pain intensity and injury severity*

| | Indirect effects | | | | Direct effects |
|---|---|---|---|---|---|
| | B | (95% CI) | p-value | % of total effect mediated | p-value |
| **Mediation via Pain Self-Efficacy** | | | | | |
| Compensation status | 0.24 | 0.07,0.41 | 0.006 | 36.9% | 0.016 |
| Fault | 0.25 | 0.09,0.41 | 0.002 | 37.7% | 0.010 |
| **Mediation via Pain Catastrophising** | | | | | |
| Compensation status[a] | n/a | n/a | n/a | n/a | n/a |
| Fault | 0.17 | 0.03,0.32 | 0.017 | 26.0% | 0.001 |
| **Mediation via Perceived Injustice** | | | | | |
| Compensation status | 0.25 | 0.10,0.40 | 0.001 | 39.1% | 0.017 |
| Fault | 0.34 | 0.19,0.48 | <0.001 | 50.4% | 0.027 |

*Notes*, all analysis adjusted only for age, sex, pain intensity and injury severity. [a] Compensation status was not associated with catastrophising, so mediation not examined.

Supplementary Table 3b

*Combined mediation effects between predictors (fault attribution and compensation status) and pain interference*

| | **Fault attribution** | **Compensation** |
|---|---|---|
| Total indirect effect | | |
| β (95% CI) | 0.08 (0.04,0.12) | 0.07 (0.03,0.10) |
| p-value | <0.001 | <0.001 |
| % of mediated | | |
| Total effect | 59.3% | 48.7% |
| Pain self-efficacy | 23.9% | 25.1% |
| Perceived injustice | 19.3% | 23.6% |
| Pain Catastrophising | 16.2% | n/a |
| Direct effect (p-value) | | |
| β (95% CI) | 0.05 (0.00, 0.11) | 0.06 (0.00,0.12) |
| p-value | 0.055 | 0.051 |

*Notes*: all analysis adjusted only for age, sex, pain intensity and injury severity.

Supplementary tables

Supplementary Table 3c

*Direct and indirect effects between predictors and health status (EQ-5D), adjusting for age, sex and injury severity*

| | Indirect effects | | | | Direct effects [a] |
|---|---|---|---|---|---|
| | B | (95% CI) | p-value | % of total effect mediated | p-value |
| **Mediation via Pain Self-Efficacy** | | | | | |
| Compensation status | -0.016 | -0.029,-0.002 | 0.022 | 34.8% | 0.121 |
| Fault | -0.020 | -0.035,-0.004 | 0.012 | 33.5% | 0.026 |
| **Mediation via Pain Catastrophising** | | | | | |
| Compensation status[a] | n/a | n/a | n/a | n/a | n/a |
| Fault | -0.014 | -0.026,-0.002 | 0.026 | 24.1% | 0.011 |
| **Mediation via Perceived Injustice** | | | | | |
| Compensation status | -0.017 | -0.034,-0.008 | 0.008 | 37.0% | 0.151 |
| Fault | -0.026 | -0.042,-0.015 | <0.001 | 46.1% | 0.070 |

*Notes*, all analysis adjusted only for age, sex, pain intensity and injury severity. [a] Compensation status was not associated with catastrophising, so mediation not examined.

Supplementary Table 3d

*Combined mediation effects between predictors (fault attribution and compensation status) and EQ-5D*

| | **Fault attribution** | **Compensation** |
|---|---|---|
| Total indirect effect | | |
| β (95% CI) | -0.068 (-0.103,-0.033) | -0.048 (-0.079,-0.016) |
| p-value | <0.001 | 0.002 |
| % of mediated | | |
| Total effect | 54.0% | 50.1% |
| Pain self-efficacy | 21.7% | 26.0% |
| Perceived injustice | 16.9% | 24.1% |
| Pain Catastrophising | 15.5% | n/a |
| Direct effect (p-value) | | |
| β (95% CI) | -0.058 (-0.130,0.014) | -0.047 (-0.130,0.035) |
| p-value | 0.114 | 0.258 |

*Notes*, all analysis adjusted only for age, sex, pain intensity and injury severity.

Supplementary tables

Supplementary Table 3e

*Direct and indirect effects between predictors and functional outcome (GOS-E), adjusting for age, sex and injury severity*

| | Indirect effects | | | | Direct effects[a] |
|---|---|---|---|---|---|
| | B | (95% CI) | p-value | % of total effect mediated | p-value |
| **Mediation via Pain Self-Efficacy** | | | | | |
| Compensation status | -0.025 | -0.055,0.006 | 0.110 | 11.8% | 0.004 |
| Fault | -0.032 | -0.062,-0.001 | 0.041 | 19.4% | 0.032 |
| **Mediation via Pain Catastrophising** | | | | | |
| Fault | -0.026 | -0.054,0.002 | 0.065 | 16.4% | 0.023 |
| **Mediation via Perceived Injustice** | | | | | |
| Compensation status | -0.050 | -0.087,-0.013 | 0.009 | 23.5% | 0.006 |
| Fault | -0.084 | -0.125,-0.043 | <0.001 | 50.9% | 0.189 |

*Notes*, all analysis adjusted only for age, sex, pain intensity and injury severity.

Supplementary Table 3f

*Combined mediation effects for the relationship between GoS-E and compensation status/fault*

| | **Fault attribution** | **Compensation** |
|---|---|---|
| **Total indirect effect** | | |
| β (95% CI) | -0.089 (-0.133,-0.045) | -0.053 (-0.091,-0.016) |
| p-value | <0.001 | 0.006 |
| % of mediated | | |
| Total effect | 55.6% | 25.1% |
| Pain self-efficacy | 5.6% | 3.8% |
| Perceived injustice | 43.5% | 21.2% |
| Pain Catastrophising | 6.5% | n/a |
| **Direct effect (p-value)** | | |
| β (95% CI) | -0.071 (-0.192,0.051) | -0.159 (-0.278,-0.041) |
| p-value | 0.255 | 0.009 |

*Notes*, all analysis adjusted only for age, sex, pain intensity and injury severity.

Supplementary tables
