# Supplementary material for: Associations between compensable injury, perceived fault and pain and disability 1 year after injury: a registry-based Australian cohort study
Source: BMJ Open. 2017 Oct 5;7(10):e017350. doi: 10.1136/bmjopen-2017-017350 (PMC5639991; doi:10.1136/bmjopen-2017-017350)
Supplement: Author's manuscript [file bmjopen-2017-017350.draft_revisions.pdf]

BMJ Open

BMJ Open is committed to open peer review. As part of this commitment we make the peer review history of every article we publish publicly available.

When an article is published we post the peer reviewers' comments and the authors' responses online. We also post the versions of the paper that were used during peer review. These are the versions that the peer review comments apply to.

The versions of the paper that follow are the versions that were submitted during the peer review process. They are not the versions of record or the final published versions. They should not be cited or distributed as the published version of this manuscript.

BMJ Open is an open access journal and the full, final, typeset and author-corrected version of record of the manuscript is available on our site with no access controls, subscription charges or pay-per-view fees (http://bmjopen.bmj.com).

If you have any questions on BMJ Open's open peer review process please email editorial.bmjopen@bmj.com

BMJ Open

# Worse pain and disability after compensable injury and external fault attribution: Associations with worse pain-related cognitions

| | |
|---|---|
| Journal: | *BMJ Open* |
| Manuscript ID | bmjopen-2017-017350 |
| Article Type: | Research |
| Date Submitted by the Author: | 18-Apr-2017 |
| Complete List of Authors: | Giummarra, Melita; Monash University, Department of Epidemiology and Preventive Medicine; Caulfield Pain Management and Research Centre,<br>Baker, Katharine; Monash University, School of Psychological Sciences<br>Ioannou, Liane; Monash University, School of Public Health & Preventive Medicine<br>Gwini, Stella; Monash University, Department of Epidemiology and Preventive Medicine<br>Gibson, Stephen; Caulfield Hospital, Caulfield Pain Management & Research Centre<br>Arnold , Carolyn ; Caulfield Hospital, Caulfield Pain Management & Research Centre<br>Ponsford, Jennie; Monash University, School of Psychological Sciences<br>Cameron, Peter; Monash University, Department of Epidemiology and Preventive Medicine |
| <b>Primary Subject Heading</b>: | Rehabilitation medicine |
| Secondary Subject Heading: | Epidemiology, Emergency medicine |
| Keywords: | Musculoskeletal Pain, Trauma, Trauma and Stressor Related Disorders, Insurance, Disability |
| | |

SCHOLARONE™
Manuscripts

**Title**: Worse pain and disability after compensable injury and external fault attribution: Associations with worse pain-related cognitions

**Submission:** BMJ Open

**Authors**
1. Melita J. Giummarra [a,b,c*]
2. Katharine S. Baker [c,d*]
3. Liane Ioannou [d]
4. Stella M. Gwini [a]
5. Stephen J. Gibson [c]
6. Carolyn A. Arnold [c,f]
7. Jennie Ponsford [d,e]
8. Peter Cameron [a]

* Authors Melita Giummarra and Katharine Baker contributed equally to the paper, and are co-first author.

**Affiliations**
a) Department of Epidemiology and Preventive Medicine, School of Public Health & Preventive Medicine, Monash University, Melbourne, Australia
b) Institute for Safety, Compensation & Recovery Research, Monash University, Victoria, Australia
c) Caulfield Pain Management and Research Centre, Caulfield Hospital, Victoria, Australia
d) School of Psychological Sciences and Monash Institute of Cognitive and Clinical Neurosciences, Monash University, Faculty of Medicine, Nursing and Health Sciences, Clayton, Victoria, Australia
e) Monash-Epworth Rehabilitation Research Centre, Epworth Hospital, Richmond, Victoria, Australia
f) Academic Board of Anaesthesia & Perioperative Medicine, School of Medicine Nursing & Health Sciences, Monash University, Clayton, VIC, Australia

**Correspondence**
Dr Melita Giummarra
School of Public Health & Preventive Medicine
553 St Kilda Road
Monash University MELBOURNE VIC 3004
Ph: 61 3 9903 0365
Fax: 61 3 9905 3948
Email: melita.giummarra@monash.edu

**Word Count:** 4,202

For peer review only

## ABSTRACT

**Objectives** Engaging with a compensation system increases the likelihood of developing pain after injury. Considering approximately three quarters of patients report chronic pain after traumatic injury it is important to understand why these patients are at greater risk of disabling pain in order to develop and implement effective, targeted interventions to attenuate the transition from acute injury to disabling chronic pain. The present study examined the development of pain and disability after compensable and non-compensable injury.

**Design** Prospective observational cohort study

**Setting** A Metropolitan Trauma Service in Melbourne, Victoria, Australia

**Participants** Participants were recruited from the Victorian State Trauma Registry and Victorian Orthopaedic Trauma Outcomes Registry. Of 732 participants who were referred to the study 82 could not be contacted, and 433 participated and were included in the final analysis.

**Outcome measures** Outcome measures included the Brief Pain Inventory, Glasgow Outcome Scale, EuroQol Five Dimensions questionnaire, Pain Catastrophizing Scale, Pain Self-Efficacy Questionnaire, Injustice Experience Questionnaire and the Tampa Scale of Kinesiophobia.

**Methods** Direct and indirect relationships (i.e., via pain-related cognitive appraisals) between compensation, fault and injury characteristics, recorded during hospital admission, and outcomes of pain severity, pain interference, general health-related quality of life and disability were examined.

**Results** Ordinal, linear and logistic regressions showed that injury severity, compensable injury and external fault attribution consistently predicted poorer outcomes: moderate-severe pain, higher pain interference, poorer health-related quality of life, and moderate-severe disability. Up to 59% of the total effects between compensable injury or external fault attribution, and disability and health outcomes was indirect (particularly via lower pain self-efficacy and higher perceived injustice).

**Conclusions** As these psychological factors may be attenuated through goal-directed, functional or psychological therapies such interventions should be offered early to injured persons at risk of chronic disabling pain to improve long-term recovery.


**Key words:** Musculoskeletal Pain; Trauma; Trauma and Stressor Related Disorders; Insurance, Disability

For peer review only

## STRENGTH AND LIMITATIONS OF THIS STUDY

- The study investigated the role of compensation, and fault and pain-related attributions in the development of disability and poor health outcomes 12-month post-injury.
- Ordinal, linear and logistic regression models were fit for the relationship between each predictor and outcome while controlling for confounders.
- Mediation analyses were conducted to determine the contribution of psychological variables to the relationship between independent variables and outcomes variables.
- A relatively high proportion of the cohort had post-secondary school education and slightly higher annual income than the national average, suggesting the cohort may represent a higher socioeconomic position than the general population and injury population.
- The study design was largely cross-sectional, and causality cannot be assumed among the outcomes.

For peer review only

## INTRODUCTION

Pain is a leading contributor to global disease burden.[1] After traumatic injury,[2] disabling pain affects one in every 3-4 persons three years later.[3 4] In particular, compensable injury paradoxically leads to worse outcomes, including chronic and disabling pain,[5] despite the fact that compensation claimants are essentially entitled to more benefits to support recovery, including healthcare and income replacement.[6-8] Several factors may explain the "compensation effect". Key mechanisms include the validation of injustice perceptions, stress from engaging with compensation systems[9] and several procedural factors, including (a) poor access to clear and timely information about compensation procedures, (b) lack of empathy or engagement in interactions, or (c) dissatisfaction with decisions on entitlements or compensation.[10 11] While the often arduous application procedures, which may include proving another person was at fault,[12] are associated with worse health outcomes and negative compensation system experience,[11] outcomes are nonetheless worse for claimants who perceive that they were not at fault even in systems that are not reliant on determinations of fault.[13] Injury outcomes are worse not only when engaging with compensation systems who perceive a lack of control over pain [4] and injustice or unfairness,[14] which may altogether negatively influence the capacity to cope with pain,[15] especially after compensable injury.

A large body of work has demonstrated that pain catastrophizing, defined as the tendency towards having an exaggerated or excessive focus on negative aspects of pain and a lack of control over pain,[16] is associated with poorer mental health and pain severity and disability.[17 18] Fear of exacerbating pain or causing re-injury (i.e., kinesiophobia) and self-efficacy appraisals also increasing the likelihood of avoiding activity,[19 20] and result in worse disability,[21] and poor quality of life.[22-25] When persistent pain or disability occur after a compensable injury, negative experience from compensation system processes may further compound cognitive appraisals of pain and injustice,[26-30] and there is evidence that injustice beliefs may play a mechanistic role in worse outcomes.[14 31] Moreover, experiencing stress when engaging with the compensation system may mechanistically

For peer review only

increase the likelihood of transitioning from acute to chronic pain due to its impacts on the stress regulation systems, thereby disrupting the capacity to process and regulate painful sensations.[32 33]

Many studies have confirmed that compensable injury increases the likelihood of developing persistent pain. However, whether persons engaging with compensation systems are more likely to develop maladaptive appraisals of pain, thereby leading to worse outcomes, is not known. This prospective observational cohort study examined the development of pain, catastrophising, kinesiophobia and self-efficacy after compensable and non-compensable injury, and examined the role of these psychological factors in the development of worse disability and poorer health 12-months after injury. We hypothesised that those with a compensable injury, and those who perceived that another was at fault, would be more likely to report severe and disabling pain, and that these outcomes would be associated with lower self-efficacy, and higher pain catastrophizing, kinesiophobia, and perceived injustice.

## METHODS

### Participants and recruitment

Participants were recruited from the Victorian State Trauma Outcomes Registry (VSTR) and the Victorian Orthopaedic Trauma Outcomes Registry (VOTOR)[34-36] 12-months after admission to hospital for traumatic injury. Participants were invited into the study by trauma registry staff at the conclusion of the 12-month registry interview if they were treated at The Alfred Hospital, one of the two major trauma services in Victoria, Australia. Only English-speaking participants aged 18-70 were eligible. Exclusion criteria were cognitive impairment as assessed qualitatively during trauma registry interview, or need for proxy.

The trauma registries comprise comprehensive details about patient demographics and injury and admission data, including trauma cause, mechanism and place, hospital admission, diagnoses and procedures. Injury and pain outcomes are then assessed through telephone interviews 12-months following injury. The present study administered additional questionnaires

For peer review only

about pain and mental health 12-months after injury. Participants recruited from VSTR had sustained major trauma, defined as (a) admission to the intensive care unit for >24 hours and mechanically ventilated; (b) significant injury to two or more body regions (i.e., an Abbreviated Injury Score (AIS, 2008 scoring criteria) of >2 in two or more body regions) or a total Injury Severity Score (ISS) greater than 12; or (c) urgent surgery for intracranial, intrathoracic or intra-abdominal injury, or fixation of pelvic or spinal fractures. Patients recruited from VOTOR had sustained orthopaedic (bone or soft tissue) injuries not related to metastatic disease resulting in admission to hospital for > 24 hours. This recruitment strategy aimed to ensure that any sources of bias could be identified through comparison with other publications of these registry patients, and reliance on patient recall or medical record review were minimised as injury and admission data were extract from the trauma registries.

**Materials and Procedures**

The study was approved by Alfred Hospital (study: 290/13) and Monash University (study: CF13/3276 - 2013001633) human research ethics committees. All participants gave informed written consent to participate in the study and for the researchers to obtain data from the trauma registries. Participants completed additional questionnaire measures either via telephone interview, online or in hardcopy after their 12-month registry interview.

Demographics and pre-injury health

Participant characteristics collected from the registries included sex, age at time of injury, education level and work status. Presence of comorbidities or other pre-existing health conditions at the time of hospital admission were determined using the International Statistical Classification of Diseases and Related Health Problems, Tenth Revision, Australian Modification (ICD-10-AM) diagnosis codes. Participants were also asked about other existing health conditions that might not have been captured at initial admission given that it is only mandatory to record diagnoses that may affect the admitted episode.

For peer review only

*Injury characteristics*

Injury data extracted from the trauma registries included Abbreviated Injury Severity (AIS) scores, injury severity scores (ISS),[37] length of stay in hospital (in days), and discharge destination (i.e., home or inpatient rehabilitation). Maximum AIS severity scores, and the number of body regions with an AIS score >2 (i.e., moderate to critical injuries) were used to reflect injury severity. Trauma place (i.e., transport, work, home or other), compensation status, and whether or not the person felt they were at fault were also recorded.

Pain and functional outcomes (12 months)

*The Brief Pain Inventory (BPI)* was used to quantify pain severity and interference of pain with various aspects of daily life on 11-point Numeric Rating Scales[38] (Cronbach α =.92 for pain severity subscale and .95 for pain interference subscale in the present cohort). Scores >4/10 were considered indicative of moderate-severe pain.[39 40] Level of disability was measured using the extended version of the *Glasgow Outcome Scale (GOS-E)* [41] which classifies patient status into one of eight categories: death, vegetative state, lower severe disability, upper severe disability, lower moderate disability, upper moderate disability, lower good recovery and upper good recovery. Disability outcome is determined from independence, work and leisure activity participation, and relationships with family and friends. The *EuroQol Five Dimensions questionnaire* (*EQ-5D)*[42] was used to measure general health outcomes relating to five domains: mobility, self-care, usual activities, pain or discomfort, and anxiety or depression. A summary score ranging from 0 to 1 was calculated using the UK indexed norms[43], where a score of 1 indicates the best health state, and 0 indicates the worst health outcome.

*Psychological mediators (12 months)*

The mediating effects of psychological outcomes related to pain were assessed using four measures: the Pain Catastrophizing Scale (PCS), Pain Self-Efficacy Questionnaire (PSEQ), Injustice Experience Questionnaire (IEQ) and the Tampa Scale of Kinesiophobia (TSK). The *PCS* measured the

For peer review only

tendency to have an exaggerated negative mindset in response to painful experiences[16]. It comprises 13 items, and respondents rated the degree to which they had certain thoughts and feelings when in pain (from 0 'not at all' to 4 'all the time'). All items were summed to create a total score (Cronbach α = .95 in the present sample). The *PSEQ*[44] is a 10-item inventory assessing how confident a person was that they can cope with their pain and accomplish the activities of daily life despite their pain. Confidence in these abilities was rated on a scale from 0 'not at all confident' to 6 'completely confident', and items were summed to create a total score (Cronbach α = .96 in the present data). The *IEQ*[45] is a 12-item questionnaire on which respondents indicate the frequency of certain thoughts from 0 'never' to 4 'all the time', reflecting blame or unfairness and irreparability of loss due to an injury, which are summed to create a total score (Cronbach α = .95 in the present data). The *TSK*[46] is a 17-item self-report measure of kinesiophobia (i.e. fear of movement or fear of re-injury from movement). A total score was calculated by summing all responses after inverting items 4, 8, 12 and 16 (Cronbach α = .84 in the present data).

**Data analytic approach**

Data were analysed with Stata statistical software version 14.0 (StataCorp 2015; College Station, Texas). Significance was set at α = 0.05, and the sample was sufficient for univariate regression (with adjustment for four covariates), and for detection of moderate bias-corrected bootstrapped mediated (indirect) effects, which required a minimum sample of 377 to 400 cases.[47] Participants with missing data were excluded from the respective analysis in a list-wise manner. The data were summarised with descriptive statistics. The relationship between continuous variables (e.g. pain interference and EQ-5D) and predictor variables (e.g. compensation status and fault attribution) were examined using linear regression. Ordinal regression was used for ordinal outcomes such as pain severity (0 = no pain, <4 = low pain, $\geq$4 = moderate-severe pain), [39 40] linear regression for continuous outcomes (i.e., pain interference and EQ-5D), and logistic regression was used for binary outcomes (i.e., "good" recovery vs moderate-severe disability). Univariable regression models were fit for the relationship between each predictor and outcome while

For peer review only

controlling for age, sex, pain severity and injury severity. Violation of the proportional odds assumption was assessed for each ordinal regression model, and effects were reported in accordance with these assumptions.

Mediation analyses examined the contribution of psychological variables (i.e., self-efficacy, catastrophizing and perceived injustice) to the relationship between the independent variables (compensation and fault status) and outcome variables (pain interference, health status and disability). Mediators were only included if they were significantly related to both the predictor (i.e. compensation or fault) and the outcome (i.e. pain interference, EQ-5D summary score, GOS-E outcome). Mediation analysis estimates adjusted for injury severity, pain severity, age and sex. Mediation was tested using the Sobel-Goodman mediation test with bootstrapping with 500 case resamples to obtain 95% confidence intervals.

## RESULTS

### Cohort overview

All participants had been admitted to hospital after traumatic injury from October 2012 to October 2014. A total of 732 patients were referred to the study during their 12-month follow-up VOTOR or VSTR registry interview. Seventy potential participants could not be contacted leaving 662 assessed for eligibility. Twelve participants were ineligible (two were deceased, seven were distressed, and three were unwell), and 97 declined to participate resulting in a sample of 433 participants (66.6% response rate).

The average time from injury to follow-up was 13.50 months (SD = 1.60 months). The participants were predominately male (74.8%), average age at time of injury was 44.8 years (SD = 14.2), and the majority of participants had completed post-secondary school education (63.9%), which is slightly higher than the general Australian population whereby 61% of persons aged 15-64 have a post-school qualification [48]. Almost two thirds had a household income greater than AUD $60,000 per annum (60.9%) 12-months after injury, which is slightly higher than the national average



For peer review only

household income of $52,000 [49]. One hundred and sixty nine participants indicated that they had been involved in a compensation claim for the injury for which they were admitted, including claims with the Victorian Transport Accident Commission (TAC; *n* = 141) or WorkSafe Victoria (*n* = 28). See Table 1 for an overview of the cohort characteristics (n = 433).

**Predictors of pain severity**

Data on pain and pain-related outcomes are summarised in Table 2. At 12 months post-injury, the majority of the sample reported pain of low severity (i.e., <4/10; n=258, 59.6%), with 63 (14.5%) reporting no pain at all, and 112 (25.9%) reporting moderate-severe pain (i.e., $\geq$4/10). There was a modest correlation between age and pain severity ($r_s$ = .13, p<.006), and females were more likely to report moderate-severe pain than males (OR: 1.71; 95% CI: 1.06, 2.75). Participants with lower education (i.e., year 11 or below) were more likely to report moderate-severe pain (OR 2.80; 95% CI: 1.55, 5.05) than those with tertiary education. Likewise, participants who were not employed prior to injury (RR 3.35, 95% CI 1.65, 6.81), or had not returned to work 12 months post injury (OR 2.95, 95% CI 1.69, 5.15), were more likely to report moderate-severe pain than those who were working before injury or returned to work.

Relationships between baseline injury characteristics and pain severity at 12-months post-injury were examined with ordinal regression, adjusting for injury severity, age, sex and education; see Table 3. Participants were more likely to have pain if they had a more severe injury, such that for each additional body region with a moderate-critically severe injury, there was a 37% increase in the odds of having moderate-severe pain 12-months after injury. Likewise, the likelihood of having moderate-severe pain was predicted by longer hospital stay (4% increased odds of worse pain for each additional day), having a compensable injury (32% increased odds of pain), and attributing fault to another (46% increased odds of pain). However, place of injury (i.e. transport, work, home or elsewhere), compensation status and fault attribution were not related to pain severity when adjusting for injury and demographic characteristics.

For peer review only

Table 1

*Cohort characteristics*

| | Category | Total N | Total % | Compensable N = 169 N | Compensable N = 169 % | Not Compensable N = 264 N | Not Compensable N = 264 % |
|---|---|---|---|---|---|---|---|
| **Demographic characteristics** | | | | | | | |
| Sex | Male | 324 | 74.8 | 128 | 75.7 | 196 | 74.2 |
| | Female | 109 | 25.2 | 41 | 24.3 | 68 | 25.8 |
| Age at injury | 18-30 | 91 | 21.3 | 41 | 24.7 | 50 | 19.1 |
| | 31-40 | 70 | 16.4 | 23 | 13.9 | 47 | 17.9 |
| | 41-50 | 82 | 19.2 | 38 | 22.9 | 44 | 16.8 |
| | 51-60 | 122 | 28.5 | 42 | 25.3 | 80 | 30.5 |
| | 61+ | 63 | 14.7 | 22 | 13.3 | 41 | 15.6 |
| Presence of >1 comorbidity | None | 274 | 63.3 | 109 | 64.5 | 165 | 62.5 |
| | >1 | 159 | 36.7 | 60 | 35.5 | 99 | 37.5 |
| Highest education | Post-school education[a] | 272 | 64.5 | 102 | 63.4 | 170 | 65.1 |
| | Completed Year 12 | 64 | 15.2 | 27 | 16.8 | 37 | 14.2 |
| | Year 11 or less | 86 | 20.4 | 32 | 19.9 | 54 | 20.7 |
| Household income (p/a at 12-months after injury) | $20-40,000 | 98 | 23.6 | 40 | 26.3 | 58 | 22.1 |
| | $41-60,000 | 64 | 15.4 | 23 | 15.1 | 41 | 15.6 |
| | $61-80,000 | 67 | 16.1 | 30 | 19.7 | 37 | 14.1 |
| | $81-100,000 | 51 | 12.3 | 19 | 12.5 | 32 | 12.2 |
| | $100,000+ | 135 | 32.5 | 40 | 26.3 | 95 | 36.1 |
| **Work characteristics** | | | | | | | |
| Employment field | White Collar | 179 | 41.3 | 56 | 35.0 | 123 | 45.1 |
| | Blue Collar | 174 | 40.2 | 76 | 47.5 | 98 | 35.9 |
| | Not Working/Studying | 80 | 18.4 | 28 | 17.5 | 52 | 19.1 |
| **Injury characteristics** | | | | | | | |
| Moderate-Critical Injury[b] | 1. Head | 120 | 27.7 | 59 | 34.9 | 61 | 23.1 |
| | 2. Face | 82 | 18.9 | 42 | 24.9 | 40 | 15.2 |
| | 3. Neck | 12 | 2.8 | 8 | 4.7 | 4 | 1.5 |
| | 4. Thorax | 146 | 33.7 | 90 | 53.3 | 56 | 21.2 |
| | 5. Abdomen | 50 | 11.5 | 39 | 23.1 | 11 | 4.2 |
| | 6. Spine | 151 | 34.9 | 65 | 38.5 | 86 | 32.6 |
| | 7. Upper Extremity | 165 | 38.1 | 77 | 45.6 | 88 | 33.3 |
| | 8. Lower Extremity | 218 | 50.3 | 100 | 59.2 | 118 | 44.7 |
| | 9. Unspecified | 35 | 8.1 | 16 | 9.5 | 19 | 7.2 |
| Discharge destination | Home | 304 | 70.2 | 92 | 54.4 | 212.0 | 80.3 |
| | Rehabilitation | 129 | 29.8 | 77 | 45.6 | 52.0 | 19.7 |

[a] Tertiary education included post-secondary school certificate, diploma, bachelor or post-graduate degree; [b] Body region with severe injury with an AIS severity score of 2-5, and multiple responses per participant were possible.

For peer review only

Table 2

*Pain and pain-related outcomes in compensable and non-compensable participants*

| | Measure | Statistic | Compensable N = 160 | | Not Compensable N = 273 | | P | Effect size |
|---|---|---|---|---|---|---|---|---|
| Pain Severity | BPI | M(SD) | 2.94 | (2.19) | 2.30 | (1.94) | .002 | .31 |
| Pain interference | BPI | M(SD) | 3.39 | (2.78) | 2.16 | (2.28) | <.001 | .48 |
| Pain catastrophising | PCS | M (Median) | 12.08 | (8.00) | 7.91 | (4.00) | <.001* | .37 |
| Pain self-efficacy | PSEQ | M(SD) | 41.41 | (15.43) | 47.78 | (13.14) | <.001 | .44 |
| Kinesiophobia | TSK | M(SD) | 38.45 | (8.39) | 36.30 | (7.99) | .008 | .26 |
| Perceived injustice | IEQ | M(SD) | 20.52 | (14.61) | 13.73 | (12.40) | <.001 | .50 |

*Notes:* Effect sizes are all Cohens d, significance tests were independent samples t-tests, or non-parametric Mann Whitney U tests for data that were not normally distributed (*)

For peer review only

Table 3

*Relationship between injury characteristics and pain severity (ordinal regression)*

| Characteristics | | No Pain N = 63 (14.5%) | Low Pain N = 258 (59.6%) | Mod-Severe Pain N = 112 (25.9%) | OR Unadj | OR[a] (95% CI) |
|---|---|---|---|---|---|---|
| **Injury severity** | | | | | | |
| AIS count[+] | M (SD) | 1.51 (0.82) | 1.75 (1.07) | 2.13 (1.33) | 1.38 | 1.37 (1.15, 1.62)* |
| **Hospital Stay (continuous)**[++] | | | | | | |
| None vs any pain | M (SD) | 6.49 (6.36) | 6.72 (8.13) | | 1.00 | 0.98 (0.95, 1.02) |
| None/low vs mod/severe pain | M (SD) | 5.69 (5.99) | | 9.53 (11.31) | 1.05* | 1.04 (1.01, 1.07)* |
| **Injury place** | | | | | | |
| At home | N (%) | 14 (22.2) | 45 (17.4) | 18 (16.1) | Ref | Ref |
| Traffic/Road | N (%) | 23 (36.5) | 96 (37.2) | 54 (48.2) | 1.52 | 1.38 (0.75, 2.52) |
| Workplace | N (%) | 4 (6.4) | 25 (9.7) | 16 (14.3) | 1.98 | 1.99 (0.93, 4.26) |
| Other | N (%) | 22 (34.9) | 92 (35.7) | 24 (21.4) | 0.88 | 1.11 (0.60, 2.06) |
| **Compensation status** | | | | | | |
| None | N (%) | 41 (65.1) | 169 (65.5) | 55 (49.1) | Ref | Ref |
| TAC/Worksafe | N (%) | 22 (34.9) | 89 (34.5) | 57 (50.9) | 1.68* | 1.32 (0.84, 2.07) |
| **Fault** | | | | | | |
| At fault | N (%) | 36 (57.1) | 133 (52.0) | 46 (41.8) | Ref | Ref |
| Not at fault | N (%) | 27 (42.9) | 123 (48.0) | 64 (58.2) | 1.50* | 1.46 (0.99, 2.15) |

*Notes*: Significant relationships are with an asterix (*). [a] adjusted for age, sex and education. Analysis of Hospital stay, injury place, compensation, fault, and work status also controlled for injury severity.

[+] AIS count = the number of mod-critical injured body regions,

[++] The proportional odds assumption was not met for length of hospital stay, so outcomes are reported here for each ordinal comparison.

For peer review only

**Predictors of psychological variables**

Figure 1 shows associations between baseline injury characteristics and psychological functioning in relation to pain at 12 months. Linear regressions showed that catastrophizing, self-efficacy and perceived injustice were all worse in those who were discharged to inpatient rehabilitation following their injury, and in those who attributed fault to another. Self-efficacy was lower in participants who had a compensable injury or a longer hospital stay. Perceived injustice was worst in participants with transport or work-related injuries compared to those with an injury at home or elsewhere, after compensable injury, and after longer hospital stay. Kinesiophobia was not related to any injury characteristics.

**Predictors of poor functional recovery**

Predictors of pain interference were examined only in participants who reported some pain 12-months after injury ($n$ = 370). Most participants reported low levels of pain interference (<4/10; n=249, 67.5%), and the remainder (n=120, 32.3%) reported moderate to severe pain interference (i.e. ≥4/10), with average pain interference of 3.04 (SD = 2.49) in participants reporting some pain.

The average EQ-5D summary scores ranged from 0.70 to 0.86 (see Supplementary Table 1), indicating moderate-to-good health outcomes. According to GOS-E scores, 210 (48.5%) participants had "good" functional recovery outcomes, 216 (49.9%) had moderate disability and seven (1.6%) patients had severe disability. Given the small number of patients who had severe disability in this cohort, the moderate and severe disability groups were combined for all subsequent analyses.

Figure 2 shows the relationship between baseline characteristics and functional outcomes from the regressions. Participants showed poorer outcomes across all three functional recovery measures (BPI Interference; EQ-5D; GOS-E) if they sustained a compensable injury, attributed fault to another and required inpatient rehabilitation (see Supplementary Materials for specific ORs and CIs). At 12-months post-injury, disability (GOS-E) was more likely in those who were *employed* prior to injury, whereas pain interference and overall health were worse in those who were *unemployed* prior to injury. Pre-existing medical conditions were not associated with any functional outcomes;

For peer review only

however, it should be noted that the sample generally had good health prior to injury, with only 132 patients (35.7%) reporting one or more comorbidities at the time of the injury, and an average rating of pre-injury health of 89.16 out of 100 (SD=10.76), where 100 indicates “best imaginable health state”.

All psychological variables (self-efficacy, kinesiophobia, catastrophizing, and perceived injustice) were predictive of poorer functional outcomes of pain interference, EQ-5D, and GOS-E disability after controlling for demographics, pain severity and injury severity (Table 4).

-- Insert Figure 1 about here --

-- Insert Figure 2 about here --

Table 4

*Association between mediators and (a) Pain interference, (b) EQ-5D, and (c) GOS-E recovery outcome.*

| | Pain Interference | | EQ-5D Summary Score | | GOS-E Functional Outcome | |
|---|---|---|---|---|---|---|
| **Mediators** | **B** | **(95% CI)** | **β** | **(95% CI)** | **OR** | **(95% CI)** |
| Pain Severity | 1.02 | 0.95,1.10 | -0.065 | -0.077,-0.053 | 0.59 | 0.52,0.68 |
| Pain Self-Efficacy | -0.06 | -0.08,-0.04 | 0.005 | 0.003,0.007 | 1.04 | 1.01,1.06 |
| Kinesiophobia | 0.07 | 0.04,0.09 | -0.004 | -0.007,-0.001 | 0.95 | 0.91,0.98 |
| Catastrophising | 0.08 | 0.06,0.10 | -0.007 | -0.010,-0.004 | 0.95 | 0.91,0.98 |
| Perceived Injustice | 0.06 | 0.04,0.07 | -0.004 | -0.006,-0.003 | 0.94 | 0.92,0.96 |

*Notes*: all analysis adjusted only for age, sex, pain severity and injury severity. The sample for pain interference regression only comprised participants reporting a pain intensity>0; N=370). Pain interference and EQ-5D summary score analysed with linear regression, GOS-E analysed with logistic regression (comparing “good” recovery vs moderate to severe disability, where higher odds indicate increased likelihood of the good outcome)

**Indirect effects on functional outcomes**

Mediation analyses showed that many of the relationships between compensation, fault, and functional outcomes were either fully or partially mediated by the psychological pain variables. The exceptions were that compensation was not associated with kinesiophobia or catastrophizing, and fault was not associated with kinesiophobia, so these variables were not included as potential mediators in the respective analyses.

For peer review only

The relationships between fault attribution and the outcomes of pain interference and health status were completely mediated by self-efficacy, perceived injustice and catastrophizing. The combined mediated effect between fault attribution and outcomes via pain self-efficacy, catastrophizing and injustice was 59.3% for pain interference, 54.0% health status and 55.6% for disability. For all three outcomes the indirect effect was significantly different from zero, see Table 5.

A similar pattern was found for the relationship between compensation and functional outcomes. Self-efficacy fully mediated the effect of compensation on pain interference, overall health and disability at 12-months post-injury, perceived injustice fully mediated the association between compensation and pain interference and disability, but only partially mediated the relationship between compensation and general health. When considered together, the proportion of the total effect between compensation and functional outcomes that was mediated by self-efficacy and perceived injustice was 48.7% for pain interference, 50.1% for overall health and 25.1% for disability. Table 5 shows that the indirect effects for all three outcomes were significantly different from zero.

For peer review only

Table 5

*Direct and indirect (mediated) effects between predictors (compensation; fault) and functional outcomes (BPI Interference; EQ-5D; GOS-E) mediated by psychological outcomes (pain self-efficacy; perceived injustice; pain catastrophizing)*

| | Pain interference (BPI) | | | Health (EQ-5D) | | | Disability (GOS-E) | | |
|---|---|---|---|---|---|---|---|---|---|
| | Indirect (mediated) effect | | Direct effect | Indirect (mediated) effect | | Direct effect | Indirect (mediated) effect | | Direct effect |
| | *B* (95% CI) | *p* | *p* | *B* (95% CI) | *p* | *p* | *B* (95% CI) | *p* | *p* |
| **Pain Self-efficacy** | | | | | | | | | |
| Compensation | 0.24 (0.07,0.41) | 0.006 | 0.016 | -0.02 (-0.29,-0.002) | 0.022 | 0.121 | -0.25 (-0.06,0.01) | 0.110 | 0.004 |
| Fault | 0.25 (0.09,0.41) | 0.002 | 0.010 | -0.02 (-0.35,-0.004) | 0.012 | 0.026 | -0.32 (-0.06,-0.001) | 0.041 | 0.032 |
| **Perceived injustice** | | | | | | | | | |
| Compensation | 0.25 (0.10,0.40) | 0.001 | 0.017 | -0.02 (-0.03,-0.01) | 0.008 | 0.151 | -0.05 ( -0.09,-0.01) | 0.009 | 0.006 |
| Fault | 0.34 (0.19,0.48) | <0.001 | 0.027 | -0.03 (-0.04,-0.02) | <0.001 | 0.070 | -0.08 (-0.13,-0.04) | <0.001 | 0.189 |
| **Catastrophizing** | | | | | | | | | |
| Fault | 0.17 (0.03,0.32) | 0.017 | 0.001 | -0.01 (-0.03,-0.002) | 0.026 | 0.011 | -0.03 (-0.05,0.002) | 0.065 | 0.023 |
| **Combined mediator effects for** | | | | | | | | | |
| Compensation | 0.07 (0.03,0.10) | <0.001 | 0.051 | -0.05 (-0.08,-0.02) | 0.002 | 0.258 | -0.05 (-0.09,-0.02) | 0.006 | 0.009 |
| Fault | 0.08 (0.04,0.12) | <0.001 | 0.055 | -0.07 (-0.10,-0.03) | <0.001 | 0.114 | -0.09 (-0.13,-0.05) | <0.001 | 0.255 |

*Note*: Analyses were univariate, adjusting for age, sex, pain severity and injury severity.

For peer review only

## DISCUSSION

This study demonstrates that characteristics at the time of injury, especially compensable injury and attributing fault to another were robustly associated with poorer health and functional outcomes, including pain-related disability. These associations were observed both before and after controlling for injury severity and demographic factors that were also associated with worse outcomes. The exception was that the development of pain after compensable injury was partly attributable to injury severity, highlighting that although we replicated the so called “compensation effect”,[5 6 50] the mechanism of injury in the majority of compensable cases in the present study (i.e., transport injury) may have played a role in the persistence of pain. Specifically, injury in motor vehicle crashes are more likely to involve high energy collisions resulting in more complex multi-trauma. Nonetheless, we show for the first time that compensable patients are more likely to also develop lower self-efficacy and higher perceptions of injustice, which seem to play a role in the development of disability and poor health 12-months after injury.

The relationship between fault and disability was found to be completely mediated by perceived injustice. Previous studies have found attributions of fault are predictive of a range of poor health outcomes.[51 52] Here we show that when adjusting for injury severity, attributions of fault lead to global perceptions of injustice and worse disability and that, although these associations are no doubt bidirectional, perceived injustice has been shown to have real and fundamental effects on rehabilitation outcomes highlighting that these complex appraisals deserve greater attention. Specifically, the harmful effects of perceived injustice begin relatively early in the disability trajectory,[53] can affect the quality of working relationship with health professionals,[54] promote behaviours that are not conducive to recovery,[14] and promote an inflexible focus on justice violations that can impede recovery.[31] In the worst case scenario, these appraisals may even lead to chronic embitterment and a range of harmful long term mental health impacts.[55] Clearly, therefore, it is important to address injustice perceptions and promote rehabilitation gains early after injury in order to support a healthy adjustment to life after injury.

For peer review only - http://bmjopen.bmj.com/site/about/guidelines.xhtml

We demonstrated an association between compensable injury and worse disability and health outcomes (i.e. in relation to mobility, self-care, activity participation, pain, and anxiety/depression), which was fully mediated by self-efficacy. This finding suggests that patients who had a compensable injury, compared with non-compensable patients, had much lower confidence in participating in activities of daily living because of persistent pain. Given that low self-efficacy is a determinant of various maladaptive behaviours, such as pain avoidance and reduced participation in work, social and physical activities,[56] these effects could contribute to long term pain-related disability.[22] Promoting self-efficacy, especially after compensable injury, is clearly a high priority in order to reduce long term disability, and to promote health-related quality of life.[24]

While pain catastrophizing was not worse after compensable injury, it was associated with pain severity and pain interference. Catastrophizing played a significant role in mediating the association between fault attributions and pain interference, health and disability, but it explained a smaller proportion of the total effects between fault and injury outcomes than self-efficacy and perceived injustice. Only a quarter of this sample developed moderate-severe pain, but just over half developed moderate to severe disability. Therefore it may be that the source of catastrophic appraisals were more specific to negative thoughts about the impacts of the injury and the sense of loss and fairness (measured by the IEQ),[15 30] than those relating to catastrophic thoughts about pain (measured by the PCS). Finally, while kinesiophobia was associated with worse functional outcomes, it was not associated with any injury characteristics, including compensation and fault attributions. Evidently fear of re-injury, or exacerbating pain, is not linearly associated with the severity of the initial injury. Rather, emerging functional and psychological impacts of the injury, together with enduring personality traits, may play a greater role in kinesiophobia than injury severity.

**Clinical implications**

It is clear that some injury and demographic characteristics increase the risk of persistent pain and disability after injury. There remains a pressing need to develop and implement effective psychosocial interventions during the subacute phase after injury, particularly after compensable

For peer review only

injury, and for patients who believe that another was at fault. Given that self-efficacy and perceived injustice were important predictors and mediators of the relationships between injury and functional outcome, further investigation is needed to understand whether these appraisals should be specifically targeted in interventions.

At this stage, research on early interventions for the prevention of pain, disability, and injustice after injury are sparse.[31 57] Interventions delivered in the acute or sub-acute stage after injury that have been shown to have positive effects on self-efficacy typically comprise education,[58] and work towards building “mastery” of activities that had become difficult because of pain, and using behavioural achievements as a catalyst for positive change (i.e. improved functional outcomes), which have been shown to be more powerful than verbal encouragement alone.[24] Disability and perceptions of injustice are strongly related.[31 45] These interventions targeting either factor appear to elicit positive effects on the other. This is particularly relevant given that rehabilitation programs that optimize function (e.g., to promote re-integration into work and activities and reduce disability) lead to reductions in injustice appraisals.[59] New interventions could be developed and trialled to modulate injustice beliefs directly, especially for persons with injuries that result in permanent disability (e.g., after spinal cord injury or brain injury). Patients with strong injustice beliefs may benefit from therapies that enhance anger management, acceptance [60] or forgiveness [61]. Ultimately, when designing any intervention to target complex psychological, pain and disability outcomes after injury it is important to bear in mind that feelings of injustice frequently extend far beyond the person at fault for causing the injury, and may be applied to the compensation system, employers, health care providers, lawyers, and society as a whole.[53 62 63] Thus it is important that therapists and policy makers take a whole of person, and whole of system, approach to supporting injury recovery. Finally, procedures involved in claiming compensation, such as access to information or interactions with claims staff, which were not measured in this study, should be evaluated to ensure that these procedures are not causing secondary harm.[10 62] Indeed,

For peer review only

compensation systems are in a valuable position whereby they can optimise their systems and client relationships to bolster client self-efficacy.

**Limitations**

Some limitations of the present study should be considered. First, the cohort had a relatively high proportion of patients had post-secondary school education (slightly higher than national incidence of post-school qualifications [48]), and a slightly higher annual income than the national average.[49] This suggests that the cohort may represent a slightly higher socioeconomic position than both the general population and injury population, which should be considered when applying these findings to the trauma population. The present study was largely cross-sectional, so we cannot assume causality among the outcomes measured. For instance, the pain-related appraisals may have been a reaction to the injury event, or have been exacerbated by experiences with compensation and/or health care providers. Nonetheless, our results suggest that these constructs are powerful indicators of future recovery alongside injury severity, and should be considered when managing or treating injured persons. Future studies are now required to examine longitudinal changes in self-efficacy and perceptions of injustice throughout the life of a compensation claim to determine whether these factors may identify which persons warrant more intensive interventions, and when.

In conclusion, the results of the present study suggest that pain is more likely after compensable injury largely because these injuries are more severe and complex. However, even when taking into consideration injury severity, compensable injury led to worse pain-related disability, self-efficacy, general health and disability. Moreover, perceived injustice and low self-efficacy appear to play a key role in the poor outcomes seen after compensable injury, and warrant further investigation as a potential target both when screening patients for risk of pain and disability, and delivering targeted therapy. Early interventions should focus on enhancing self-efficacy, especially in those engaging with compensation a system.

For peer review only

## FOOTNOTES

**Acknowledgements:** We acknowledge the contribution of Nellie Georgiou-Karistianis and Paul Jennings in the general design of the study, Melissa Hart and Mimi Morgan for participant recruitment, Susan McLellan for data extraction and injury coding, Amy Allen, Jessica Frisina and Samantha Finan for data collection.

**Contributors:** Each co-author contributed significantly to the work described and commented on the manuscript. MJG designed and supervised the conduct of the study, contributed to data preparation and analysis, discussed the results, and co-wrote the manuscript with guidance and feedback from the co-authors. KSB assisted with patient recruitment and data collection, prepared data for analysis, and contributed to interpretation and writing of the manuscript. LI assisted with patient recruitment and data collection, preparation of data for analysis, interpretation and writing of the manuscript. SMG assisted with data analysis, interpretation of findings, and writing and editing of the manuscript. SJG, CAA, JP and PC assisted in designing the project and analysis, interpretation of the findings, and review of the manuscript.

**Funding:** This research was supported by an Australian Research Council (ARC) Linkage Grant [LP120200033] in collaboration with the Victorian Transport Accident Commission (TAC), and a Platform Access Grant from Monash University [PAG15-0026]. MJG was supported by an NHMRC early career fellowship [APP1036124], and PC was supported by a NHMRC fellowship [APP545926]. The Victorian Orthopaedic Trauma Outcomes Registry (VOTOR) is funded by the Transport Accident Commission via the Institute for Safety, Compensation and Recovery Research (ISCRR). The Victorian State Trauma Registry (VSTR) is a Department of Health, State Government of Victoria and Transport Accident Commission funded project. The Victorian State Trauma Outcome Registry and Monitoring (VSTORM) group is thanked for the provision of VSTR data. The funders were consulted throughout the design of the study, but played no role in data collection and analysis, the decision of how and what to publish, or the preparation of this manuscript.

**Competing interests:** None declared.

**Ethics approval:** Alfred Hospital (study: 290/13) and Monash University (study: CF13/3276 – 2013001633) human research ethics committees.

**Data sharing statement:** The Victorian State Trauma Registry (VSTR) and the Victorian Orthopaedic Trauma Outcomes Registry (VOTOR) have strict data/publication policies and guidelines to protect against unauthorized use or misinterpretation of the data. Data is only available upon request from the corresponding author (MJG).

For peer review only

## REFERENCES

1. Murray CJ, Vos T, Lozano R, et al. Disability-adjusted life years (DALYs) for 291 diseases and injuries in 21 regions, 1990–2010: a systematic analysis for the Global Burden of Disease Study 2010. *The Lancet* 2012;380(9859):2197-223. doi: 10.1016/S0140-6736(12)61689-4
2. O'Donnell ML, Varker T, Holmes AC, et al. Disability after injury: the cumulative burden of physical and mental health. *The Journal of clinical psychiatry* 2013;74(2):e137-43. doi: 10.4088/JCP.12m08011
3. Holmes AC, Williamson O, Hogg M, et al. Determinants of chronic pain 3 years after moderate or serious injury. *Pain medicine* 2013;14(3):336-44.
4. Holmes AC, Williamson O, Hogg M, et al. Predictors of pain 12 months after serious injury. *Pain medicine* 2010;11(11):1599-611.
5. Giummarra MJ, Ioannou L, Ponsford J, et al. Chronic pain following motor vehicle collision: A systematic review of outcomes associated with seeking or receiving compensation. *The Clinical journal of pain* 2016;in press, DOI: 10.1097/AJP.0000000000000342
6. Rasmussen C, Leboeuf-Yde C, Hestbaek L, et al. Poor outcome in patients with spine-related leg or arm pain who are involved in compensation claims: a prospective study of patients in the secondary care sector. *Scandinavian journal of rheumatology* 2008;37(6):462-8. doi: 10.1080/03009740802241709
7. Harris I, Young J, Jalaludin BB, et al. Predictors of neck pain after motor vehicle collisions: A prospective survey. *Journal of Orthopaedic Surgery* 2011;19(3):317-21.
8. Cassidy JD, Carroll LJ, Cote P, et al. Effect of eliminating compensation for pain and suffering on the outcome of insurance claims for whiplash injury. *New England Journal of Medicine* 2000;342(16):1179-86.
9. O'Donnell ML, Grant G, Alkemade N, et al. Compensation seeking and disability after injury: the role of compensation-related stress and mental health. *The Journal of clinical psychiatry* 2015;76(8):e1000-5. doi: 10.4088/JCP.14m09211
10. Kilgour E, Kosny A, Akkermans A, et al. Procedural Justice and the Use of Independent Medical Evaluations in Workers' Compensation. *Psychological Injury and Law* 2015;8(2):153-68. doi: 10.1007/s12207-015-9222-6
11. Ioannou L, Braaf S, Cameron P, et al. Compensation System Experience at 12 Months After Road or Workplace Injury in Victoria, Australia. *Psychological Injury and Law* 2016;9(4):376-89. doi: 10.1007/s12207-016-9275-1
12. Elbers NA, Collie A, Hogg-Johnson S, et al. Differences in perceived fairness and health outcomes in two injury compensation systems: a comparative study. *BMC public health* 2016;16:658. doi: 10.1186/s12889-016-3331-3
13. Gabbe BJ, Cameron PA, Williamson OD, et al. The relationship between compensable status and long-term patient outcomes following orthopaedic trauma. *Medical Journal of Australia* 2007;187(1):14-17.
14. Sullivan MJL, Davidson N, Garfinkel B, et al. Perceived Injustice is Associated with Heightened Pain Behavior and Disability in Individuals with Whiplash Injuries. *Psychological Injury and Law* 2009;2(3-4):238-47. doi: 10.1007/s12207-009-9055-2
15. Sullivan MJL, Adams H, Martel MO, et al. Catastrophizing and perceived injustice: risk factors for the transition to chronicity after whiplash injury. *Spine* 2011;36(25 Suppl):S244-9. doi: 10.1097/BRS.0b013e3182387fed
16. Sullivan MJL, Bishop SR, Pivik J. The pain catastrophizing scale: Development and validation. *Psychological Assessment* 1995;7(4):524-32. doi: 10.1037/1040-3590.7.4.524
17. Lindal E, Breivik H. Fear and catastrophizing thoughts aggravate risks of chronic pain after a fracture. *Scandinavian Journal of Pain* 2010;1(1):7. doi: 10.1016/j.sjpain.2009.09.007
18. Gopinath B, Jagnoor J, Nicholas M, et al. Presence and predictors of persistent pain among persons who sustained an injury in a road traffic crash. *European journal of pain* 2015;19(8):1111-8. doi: 10.1002/ejp.634

For peer review only

19. Vlaeyen JW, Linton SJ. Fear-avoidance and its consequences in chronic musculoskeletal pain: A state of the art. *Pain* 2000;85(3):317-32.
20. Vlaeyen JW, Linton SJ. Fear-avoidance model of chronic musculoskeletal pain: 12 years on. *Pain* 2012;153(6):1144-7. doi: 10.1016/j.pain.2011.12.009
21. Picavet HSJ. Pain Catastrophizing and Kinesiophobia: Predictors of Chronic Low Back Pain. *American Journal of Epidemiology* 2002;156(11):1028-34. doi: 10.1093/aje/kwf136
22. Arnstein P, Caudill M, Mandle CL, et al. Self-efficacy as a mediator of the relationship between pain intensity, disability and depression in chronic pain patients. *Pain* 1999;80(3):483-91.
23. Turner JA, Ersek M, Kemp C. Self-efficacy for managing pain is associated with disability, depression, and pain coping among retirement community residents with chronic pain. *The journal of pain : official journal of the American Pain Society* 2005;6(7):471-9. doi: 10.1016/j.jpain.2005.02.011
24. Connolly FR, Aitken LM, Tower M. An integrative review of self-efficacy and patient recovery post acute injury. *Journal of advanced nursing* 2014;70(4):714-28. doi: 10.1111/jan.12237
25. Middleton J, Tran Y, Craig A. Relationship between quality of life and self-efficacy in persons with spinal cord injuries. *Archives of physical medicine and rehabilitation* 2007;88(12):1643-8. doi: 10.1016/j.apmr.2007.09.001
26. Trost Z, Agtarap S, Scott W, et al. Perceived injustice after traumatic injury: Associations with pain, psychological distress, and quality of life outcomes 12 months after injury. *Rehabilitation psychology* 2015;60(3):213-21. doi: 10.1037/rep0000043
27. Trost Z, Monden KR, Buelow M, et al. Perceived Injustice Predicts Intention to Litigate: Findings from a Spinal Cord Injury Sample. *Psychological Injury and Law* 2016;9(1):31-40. doi: 10.1007/s12207-015-9245-z
28. Yakobov E, Scott W, Stanish W, et al. The role of perceived injustice in the prediction of pain and function after total knee arthroplasty. *Pain* 2014;155(10):2040-6. doi: 10.1016/j.pain.2014.07.007
29. Sullivan MJL, Thibault P, Simmonds MJ, et al. Pain, perceived injustice and the persistence of post-traumatic stress symptoms during the course of rehabilitation for whiplash injuries. *Pain* 2009;145(3):325-31. doi: 10.1016/j.pain.2009.06.031
30. Sullivan MJL, Scott W, Trost Z. Perceived injustice: A risk factor for problematic pain outcomes. *The Clinical journal of pain* 2012;8(6):484-88.
31. Monden KR, Trost Z, Scott W, et al. The unfairness of it all: Exploring the role of injustice appraisals in rehabilitation outcomes. *Rehabilitation psychology* 2016;61(1):44-53. doi: 10.1037/rep0000075
32. McLean SA, Clauw DJ, Abelson JL, et al. The development of persistent pain and psychological morbidity after motor vehicle collision: integrating the potential role of stress response systems into a biopsychosocial model. *Psychosomatic medicine* 2005;67(5):783-90. doi: 10.1097/01.psy.0000181276.49204.bb
33. Sterling M, Hendrikz J, Kenardy J. Compensation claim lodgement and health outcome developmental trajectories following whiplash injury: a prospective study *Pain* 2010 150 22 - 8
34. Cameron PA, Gabbe BJ, McNeil JJ, et al. The trauma registry as a statewide quality improvement tool. *Journal of Trauma and Acute Care Surgery* 2005;59(6):1469-76.
35. Urquhart DM, Edwards ER, Graves SE, et al. Characterisation of orthopaedic trauma admitted to adult Level 1 Trauma Centres. *Injury-Int J Care Inj* 2006;37(2):120-27. doi: 10.1016/j.injury.2005.10.016
36. Williamson OD, Epi GDC, Gabbe BJ, et al. Predictors of moderate or severe pain 6 months after orthopaedic injury: a prospective cohort study. *Journal of orthopaedic trauma* 2009;23(2):139-44. doi: 10.1097/BOT.0b013e3181962e29
37. Baker SP, O'Neill B, Haddon WJ, et al. The Injury Severity Score: a method for describing patients with multiple injuries and evaluating emergency care. *Journal of Trauma* 1974;14:187-96.


For peer review only

38. Cleeland CS, Ryan KM. Pain assessment: global use of the Brief Pain Inventory. *Annals of the Academy of Medicine, Singapore* 1994;23(2):129-38.
39. Gerbershagen HJ, Rothaug J, Kalkman CJ, et al. Determination of moderate-to-severe postoperative pain on the numeric rating scale: a cut-off point analysis applying four different methods. *British journal of anaesthesia* 2011;107(4):619-26. doi: 10.1093/bja/aer195
40. Kapstad H, Hanestad BR, Langeland N, et al. Cutpoints for mild, moderate and severe pain in patients with osteoarthritis of the hip or knee ready for joint replacement surgery. *BMC musculoskeletal disorders* 2008;9:55. doi: 10.1186/1471-2474-9-55
41. Wilson JT, Pettigrew LE, Teasdale GM. Structured interviews for the Glasgow Outcome Scale and the extended Glasgow Outcome Scale: guidelines for their use. *Journal of neurotrauma* 1998;15(8):573-85. doi: 10.1089/neu.1998.15.573 [published Online First: 1998/09/03]
42. Herdman M, Gudex C, Lloyd A, et al. Development and preliminary testing of the new five-level version of EQ-5D (EQ-5D-5L). *Quality of Life Research* 2011;20(10):1727-36. doi: 10.1007/s11136-011-9903-x
43. Dolan P. Modeling valuations for EuroQol health states. *Medical Care* 1997;35:1095-108.
44. Nicholas MK. The pain self-efficacy questionnaire: Taking pain into account. *European journal of pain* 2007;11(2):153-63.
45. Sullivan MJL, Adams H, Horan S, et al. The role of perceived injustice in the experience of chronic pain and disability: scale development and validation. *Journal of occupational rehabilitation* 2008;18(3):249-61. doi: 10.1007/s10926-008-9140-5
46. Miller RP, Kori S, Todd D. The Tampa Scale: a measure of kinesiophobia. *The Clinical journal of pain* 1991;7(1):51-52.
47. Fritz MS, Mackinnon DP. Required sample size to detect the mediated effect. *Psychol Sci* 2007;18(3):233-9. doi: 10.1111/j.1467-9280.2007.01882.x
48. ABS. Qualifications and Work, Australia, 2015 (Cat. 4235.0). Canberra, Australia: Australian Bureau of Statistics, 2016.
49. ABS. Household Income and Wealth, Australia, 2013-14 (Cat 6523.0). Canberra, Australia: Australian Bureau of Statistics, 2015.
50. Murgatroyd DF, Cameron ID, Harris IA. Understanding the effect of compensation on recovery from severe motor vehicle crash injuries: a qualitative study. *Injury prevention : journal of the International Society for Child and Adolescent Injury Prevention* 2011;17(4):222-7. doi: 10.1136/ip.2010.029546
51. Thompson J, Berk M, O'Donnell M, et al. Attributions of responsibility and recovery within a no-fault insurance compensation system. *Rehabilitation psychology* 2014;59(3):247-55. doi: 10.1037/a0036543
52. Gabbe BJ, Simpson PM, Cameron PA, et al. Association between perception of fault for the crash and function, return to work and health status 1 year after road traffic injury: A registry-based cohort study. *BMJ Open* 2015;5(11):e009907. doi: 10.1136/bmjopen-2015009907
10.1136/bmjopen-2015-009907
53. Tait RC, Chibnall JT. Workers' Compensation Claimants with Low Back Pain: the Role of Dissatisfaction in the Transition to Disability. *Psychological Injury and Law* 2015;9(1):16-22. doi: 10.1007/s12207-015-9243-1
54. Scott W, Milioto M, Trost Z, et al. The relationship between perceived injustice and the working alliance: a cross-sectional study of patients with persistent pain attending multidisciplinary rehabilitation. *Disability and rehabilitation* 2016;38(24):2365-73. doi: 10.3109/09638288.2015.1129444
55. Sensky T. Chronic embitterment and organisational justice. *Psychotherapy and psychosomatics* 2010;79(2): 65-72. doi: doi: 10.1159/000270914
56. Asghari A, Nicholas MK. Pain self-efficacy beliefs and pain behaviour. A prospective study. *Pain* 2001;94(1):85-100.

For peer review only

57. De Silva M, Maclachlan M, Devane D, et al. Psychosocial interventions for the prevention of disability following traumatic physical injury. *The Cochrane database of systematic reviews* 2009(4):CD006422. doi: 10.1002/14651858.CD006422.pub3
58. Wong EM, Chan SW, Chair SY. Effectiveness of an educational intervention on levels of pain, anxiety and self-efficacy for patients with musculoskeletal trauma. *Journal of advanced nursing* 2010;66(5):1120-31. doi: 10.1111/j.1365-2648.2010.05273.x [published Online First: 2010/03/27]
59. Yakobov E, Scott W, Thibault P, et al. Treatment-Related Reductions in Disability Are Associated with Reductions in Perceived Injustice Following Treatment of Whiplash Injury. *Psychological Injury and Law* 2016;9(1):41-47. doi: 10.1007/s12207-015-9248-9
60. Scott W, Trost Z, Bernier E, et al. Anger differentially mediates the relationship between perceived injustice and chronic pain outcomes. *Pain* 2013;154(9):1691-8. doi: 10.1016/j.pain.2013.05.015
61. Wade NG, Worthington EL. In Search of a Common Core: A Content Analysis of Interventions to Promote Forgiveness. *Psychotherapy: Theory, Research, Practice, Training* 2005;42(2):160-77. doi: 10.1037/0033-3204.42.2.160
62. Sullivan MJL, Yakobov E, Scott W, et al. Perceived Injustice and Adverse Recovery Outcomes. *Psychological Injury and Law* 2014;7(4):325-34. doi: 10.1007/s12207-014-9209-8
63. Scott W, McEvoy A, Garland R, et al. Sources of injustice among individuals with persistent pain following musculoskeletal injury. *Psychological Injury and Law* 2016;9(1):6-15. doi: 10.1007/s12207-015-9249-8

For peer review only

## FIGURE CAPTIONS

Figure 1.

Regression beta weights and ORs for the association between injury characteristics and psychological variables of pain catastrophizing, pain self-efficacy, kinesiophobia and injustice experience, adjusted for age, sex and injury severity. Error bars (95% CI) that do not cross the central line indicate significant relationships. Tables of specific values can be found in Supplementary Materials.

Figure 2.

Regression for association between baseline characteristics and functional recovery outcomes of pain interference (only for those with pain severity>0; N=370), EQ-5D, and GOS-E, adjusted for age, sex and injury severity. Error bars (95% CI) that do not cross the central line indicate significant relationships.

For peer review only

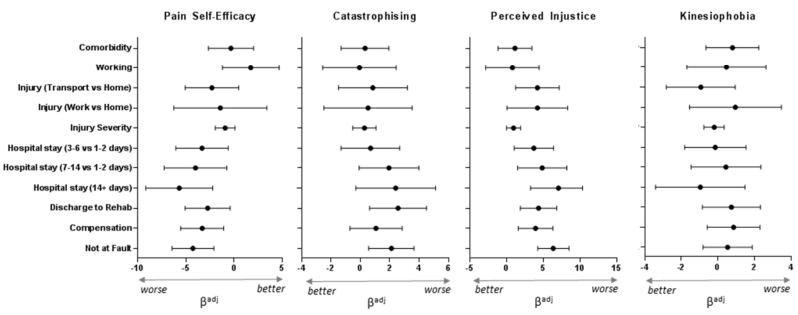


Regression beta weights and ORs for the association between injury characteristics and psychological variables of pain catastrophizing, pain self-efficacy, kinesiophobia and injustice experience, adjusted for age, sex and injury severity. Error bars (95% CI) that do not cross the central line indicate significant relationships. Tables of specific values can be found in Supplementary Materials.

338x190mm (96 x 96 DPI)

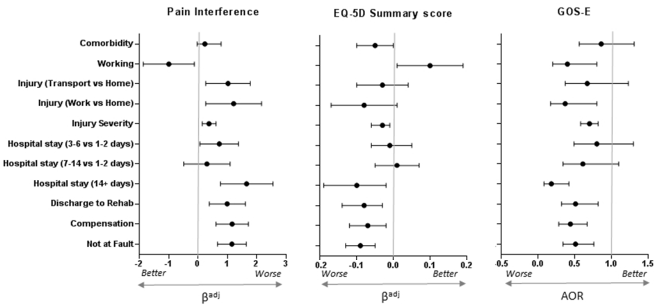


Regression for association between baseline characteristics and functional recovery outcomes of pain interference (only for those with pain severity>0; N=370), EQ-5D, and GOS-E, adjusted for age, sex and injury severity. Error bars (95% CI) that do not cross the central line indicate significant relationships.

338x190mm (96 x 96 DPI)

For peer review only

## SUPPLEMENTARY

**Supplementary Table 1**
*Multiple regression for association between injury characteristics and pain interference (only for pain intensity>0; N=370), EQ-5D, and GOS-E recovery outcome.*

| | | Pain Interference | | | | EQ-5D Summary Score | | | | GOS-E Functional Outcome | | | |
|---|---|---|---|---|---|---|---|---|---|---|---|---|---|
| **Injury characteristics** | | **M** | **(sd)** | **β** | **(95% CI)** | **M** | **(sd)** | **β** | **(95% CI)** | **N** | **%** | **OR** | **(95% CI)** |
| **Comorbidities** | None | 2.91 | 2.42 | Ref | | 0.82 | 0.22 | Ref | | 137 | 50.6 | Ref | |
| | Present | 3.27 | 2.62 | 0.23 | -0.0.32,0.78 | 0.77 | 0.25 | -0.05 | -0.10,0 | 73 | 47.1 | 0.86 | 0.56,1.31 |
| **Work status at injury** | Not working | 3.97 | 2.87 | Ref | | 0.72 | 0.33 | Ref | | 37 | 68.5 | Ref | |
| | Working | 2.91 | 2.41 | **-1.00** | **-1.87,-0.13** | 0.82 | 0.21 | **0.10** | **0.01,0.19** | 173 | 46.5 | **0.40** | **0.20,0.80** |
| **Injury place** | At home | 2.48 | 2.39 | Ref | | 0.82 | 0.24 | Ref | | 44 | 57.9 | Ref | |
| | Traffic/Road | 3.60 | 2.61 | **1.02** | **0.26, 1.78** | 0.77 | 0.25 | -0.03 | -0.10,0.04 | 66 | 39.5 | 0.67 | 0.37,1.23 |
| | Workplace | 3.60 | 2.38 | **1.21** | **0.26, 2.16** | 0.74 | 0.25 | -0.08 | -0.17,0.01 | 14 | 31.1 | **0.37** | **0.17,0.80** |
| | Other | 2.43 | 2.24 | 0.15 | -0.62, 0.92 | 0.86 | 0.18 | 0.04 | -0.03,0.10 | 86 | 62.3 | 1.37 | 0.74,2.26 |
| **Injury severity** | AIS region count | | | **0.37** | **0.14, 0.61** | | | **-0.03** | **-0.06,-0.01** | | | **0.70** | **0.58,0.82** |
| **Hospital stay** | 1-2 days | 2.48 | 2.35 | Ref | | 0.83 | 0.24 | Ref | | 95 | 60.5 | Ref | |
| | 3-6 days | 3.26 | 2.46 | **0.72** | **0.06, 1.37** | 0.81 | 0.22 | -0.01 | -0.06,0.05 | 69 | 52.3 | 0.80 | 0.49,1.30 |
| | 7-13 days | 2.88 | 2.52 | 0.30 | -0.49, 1.09 | 0.82 | 0.19 | 0.01 | -0.05,0.07 | 35 | 43.8 | 0.61 | 0.34,1.10 |
| | ≥ 14 days | 4.34 | 2.49 | **1.65** | **0.76, 2.55** | 0.70 | 0.27 | **-0.10** | **-0.19,-0.02** | 10 | 17.9 | **0.18** | **0.08,0.42** |
| **Type of discharge** | Home | 2.65 | 2.29 | Ref | | 0.84 | 0.22 | Ref | | 168 | 55.6 | Ref | |
| | Rehabilitation | 3.91 | 2.72 | **0.99** | **0.38, 1.61** | 0.73 | 0.25 | **-0.08** | **-0.14,-0.03** | 42 | 33.9 | **0.51** | **0.32,0.82** |

For peer review only

Supplementary tables

| | | | | | | | | | | | | | | |
|---|---|---|---|---|---|---|---|---|---|---|---|---|---|---|
| **Compensation status** | None | 2.53 | 2.32 | Ref | | 0.84 | 0.22 | Ref | | 155 | 58.7 | Ref | |
| | TAC/Worksafe | 3.82 | 2.57 | **1.16** | **0.61, 1.71** | 0.75 | 0.25 | **-0.07** | **-0.12,-0.02** | 55 | 34 | **0.44** | **0.28,0.67** |
| **Fault** | At fault | 2.42 | 2.14 | Ref | | 0.85 | 0.19 | Ref | | 124 | 57.9 | Ref | |
| | Not at fault | 3.63 | 2.68 | **1.15** | **0.66, 1.64** | 0.76 | 0.26 | **-0.09** | **-0.13,-0.05** | 83 | 39.9 | **0.51** | **0.34,0.76** |

*Notes*: all analysis adjusted for age, sex and injury severity, except for the injury severity analysis which only adjusted for age and sex.

For peer review only

Supplementary tables

For peer review only

Supplementary tables

Supplementary Table 2

*Multiple regression for association between injury characteristics, pain catastrophising, pain self-efficacy, kinesiophobia and injustice experience.*

| | | PCS | | | | PSEQ | | | |
|---|---|---|---|---|---|---|---|---|---|
| **Injury characteristics** | | **M** | **(sd)** | **β**** | **(95% CI)** | **M** | **(sd)** | **β**** | **(95% CI)** |
| **Comorbidities** | None | 9.54 | 11.23 | Ref | | 45.63 | 14.19 | Ref | |
| | Present | 9.30 | 10.6 | 0.32 | -1.31,1.94 | 45.02 | 14.68 | -0.34 | -2.68,2.01 |
| **Work status at injury** | Not working | 11.65 | 12.61 | Ref | | 40.99 | 15.12 | Ref | |
| | Working | 9.12 | 10.71 | -0.06 | -2.57,2.44 | 46.63 | 14.46 | 1.71 | -1.22,4.65 |
| **Injury place** | At home | 8.18 | 10.67 | Ref | | 47.41 | 12.01 | Ref | |
| | Traffic/Road | 11.38 | 11.81 | 0.85 | -1.49,3.20 | 42.7 | 15.27 | -2.32 | -5.09,0.46 |
| | Workplace | 11.13 | 11.3 | 0.52 | -2.48,3.53 | 43.11 | 15.41 | -1.44 | -6.26,3.38 |
| | Other | 7.18 | 9.49 | -1.27 | -3.55,1.00 | 48.43 | 13.37 | -0.10 | -2.97,2.76 |
| **Injury severity** | AIS region count | | | 0.28 | -0.51,1.06 | | | -0.94 | -1.96,0.08 |
| **Hospital stay** | 1-2 days | 7.91 | 10.00 | Ref | | 49.11 | 12.57 | Ref | |
| | 3-6 days | 9.77 | 11.3 | 0.69 | -1.31,2.68 | 43.81 | 14.99 | **-3.34** | **-6.05,-0.63** |
| | 7-13 days | 9.24 | 10.55 | 1.94 | -0.09,3.98 | 45.33 | 13.96 | **-4.02** | **-7.27,-0.77** |
| | ≥ 14 days | 13.25 | 12.76 | 2.40 | -0.31,5.11 | 38.89 | 15.5 | **-5.70** | **-9.16,-2.23** |
| **Type of discharge** | Home | 7.92 | 9.67 | Ref | | 47.41 | 13.61 | Ref | |
| | Rehabilitation | 13.08 | 12.97 | **2.56** | **0.63,4.50** | 40.71 | 15.01 | **-2.76** | **-5.10,-0.42** |
| **Compensation status** | None | 8.04 | 10.12 | Ref | | 47.91 | 13.29 | Ref | |
| | TAC/Worksafe | 11.67 | 11.93 | 1.06 | -0.72,2.84 | 41.43 | 15.12 | **-3.32** | **-5.55,-1.10** |

| | | | | | | | | | |
|---|---|---|---|---|---|---|---|---|---|
| **Fault** | At fault | 7.22 | 9.72 | Ref | | 48.94 | 12.07 | Ref | |
| | Not at fault | 11.67 | 11.80 | **2.11** | **0.57,3.65** | 41.87 | 15.63 | **-4.29** | **-6.45,-2.11** |

*Notes*: all analysis adjusted for age, sex and injury severity, except for the injury severity analysis which only adjusted for age and sex.

For peer review only
Supplementary tables

(Supplementary Table 2 continued)

| Injury characteristics | | TSK | | | | IEQ | | | |
|---|---|---|---|---|---|---|---|---|---|
| | | M | (sd) | β** | (95% CI) | M | (sd) | β** | (95% CI) |
| **Comorbidities** | None | 36.99 | 8.32 | Ref | | 15.99 | 13.6 | Ref | |
| | Present | 37.32 | 7.99 | 0.80 | -0.65,2.24 | 16.67 | 13.75 | 1.14 | -1.17,3.46 |
| **Work status at injury** | Not working | 37.81 | 9.33 | Ref | | 17.68 | 14.27 | Ref | |
| | Working | 37 | 8.01 | 0.47 | -1.70,2.64 | 16.02 | 13.55 | 0.80 | -2.83,4.42 |
| **Injury place** | At home | 36.97 | 7.86 | Ref | | 12.57 | 11.06 | Ref | |
| | Traffic/Road | 37.53 | 8.05 | -0.93 | -2.82,0.95 | 19.22 | 14.43 | **4.20** | **1.25,7.16** |
| | Workplace | 39.76 | 7.63 | 0.96 | -1.55,3.48 | 19.39 | 13.6 | **4.20** | **0.08,8.32** |
| | Other | 35.79 | 8.56 | -1.78 | -3.85,0.29 | 13.51 | 12.97 | 1.03 | -2.17,4.24 |
| **Injury severity** | AIS region count | | | -0.20 | -0.76,0.35 | | | 0.95 | -0.02,1.91 |
| **Hospital stay** | 1-2 days | 36.66 | 7.77 | Ref | | 12.54 | 12.57 | Ref | |
| | 3-6 days | 37.45 | 8.62 | -0.14 | -1.82,1.54 | 17.41 | 13.82 | **3.71** | **1.04,6.37** |
| | 7-13 days | 36.98 | 8.1 | 0.44 | -1.47,2.36 | 16.81 | 13.11 | **4.84** | **1.50,8.19** |
| | ≥ 14 days | 37.77 | 8.64 | -0.96 | -3.41,1.49 | 22.55 | 14.14 | **7.05** | **3.26,10.34** |
| **Type of discharge** | Home | 36.52 | 8.14 | Ref | | 14.09 | 12.87 | Ref | |
| | Rehabilitation | 38.53 | 8.18 | 0.74 | -0.85,2.33 | 21.31 | 14.12 | **4.35** | **1.88,6.81** |
| **Compensation status** | None | 36.3 | 8.05 | Ref | | 13.66 | 12.51 | Ref | |
| | TAC/Worksafe | 38.37 | 8.28 | 0.86 | -0.58,2.31 | 20.31 | 14.39 | **3.95** | **1.60,6.31** |

For peer review only
Supplementary tables

| | | | | | | | | | |
|---|---|---|---|---|---|---|---|---|---|
| **Fault** | At fault | 36.19 | 7.79 | Ref | | 11.94 | 11.1 | Ref | |
| | Not at fault | 38.03 | 8.54 | 0.54 | -0.81,1.89 | 20.5 | 14.62 | **6.35** | **4.23,8.48** |

*Notes*: all analysis adjusted for age, sex, pain intensity and injury severity.

For peer review only
Supplementary tables

Supplementary Table 3a

*Direct and indirect effects between predictors and pain interference, adjusting for age, sex, pain intensity and injury severity*

| | Indirect effects | | | | Direct effects |
|---|---|---|---|---|---|
| | B | (95% CI) | p-value | % of total effect mediated | p-value |
| **Mediation via Pain Self-Efficacy** | | | | | |
| Compensation status | 0.24 | 0.07,0.41 | 0.006 | 36.9% | 0.016 |
| Fault | 0.25 | 0.09,0.41 | 0.002 | 37.7% | 0.010 |
| **Mediation via Pain Catastrophising** | | | | | |
| Compensation status[a] | n/a | n/a | n/a | n/a | n/a |
| Fault | 0.17 | 0.03,0.32 | 0.017 | 26.0% | 0.001 |
| **Mediation via Perceived Injustice** | | | | | |
| Compensation status | 0.25 | 0.10,0.40 | 0.001 | 39.1% | 0.017 |
| Fault | 0.34 | 0.19,0.48 | <0.001 | 50.4% | 0.027 |

*Notes*, all analysis adjusted only for age, sex, pain intensity and injury severity. [a] Compensation status was not associated with catastrophising, so mediation not examined.

Supplementary Table 3b

*Combined mediation effects between predictors (fault attribution and compensation status) and pain interference*

| | **Fault attribution** | **Compensation** |
|---|---|---|
| Total indirect effect | | |
| β (95% CI) | 0.08 (0.04,0.12) | 0.07 (0.03,0.10) |
| p-value | <0.001 | <0.001 |
| % of mediated | | |
| Total effect | 59.3% | 48.7% |
| Pain self-efficacy | 23.9% | 25.1% |
| Perceived injustice | 19.3% | 23.6% |
| Pain Catastrophising | 16.2% | n/a |
| Direct effect (p-value) | | |
| β (95% CI) | 0.05 (0.00, 0.11) | 0.06 (0.00,0.12) |
| p-value | 0.055 | 0.051 |

*Notes*: all analysis adjusted only for age, sex, pain intensity and injury severity.

For peer review only

Supplementary tables

Supplementary Table 3c
*Direct and indirect effects between predictors and health status (EQ-5D), adjusting for age, sex and injury severity*

| | Indirect effects | | | | Direct effects [a] |
|---|---|---|---|---|---|
| | B | (95% CI) | p-value | % of total effect mediated | p-value |
| **Mediation via Pain Self-Efficacy** | | | | | |
| Compensation status | -0.016 | -0.029,-0.002 | 0.022 | 34.8% | 0.121 |
| Fault | -0.020 | -0.035,-0.004 | 0.012 | 33.5% | 0.026 |
| **Mediation via Pain Catastrophising** | | | | | |
| Compensation status[a] | n/a | n/a | n/a | n/a | n/a |
| Fault | -0.014 | -0.026,-0.002 | 0.026 | 24.1% | 0.011 |
| **Mediation via Perceived Injustice** | | | | | |
| Compensation status | -0.017 | -0.034,-0.008 | 0.008 | 37.0% | 0.151 |
| Fault | -0.026 | -0.042,-0.015 | <0.001 | 46.1% | 0.070 |

*Notes*, all analysis adjusted only for age, sex, pain intensity and injury severity. [a] Compensation status was not associated with catastrophising, so mediation not examined.

Supplementary Table 3d
*Combined mediation effects between predictors (fault attribution and compensation status) and EQ-5D*

| | **Fault attribution** | **Compensation** |
|---|---|---|
| Total indirect effect | | |
| β (95% CI) | -0.068 (-0.103,-0.033) | -0.048 (-0.079,-0.016) |
| p-value | <0.001 | 0.002 |
| % of mediated | | |
| Total effect | 54.0% | 50.1% |
| Pain self-efficacy | 21.7% | 26.0% |
| Perceived injustice | 16.9% | 24.1% |
| Pain Catastrophising | 15.5% | n/a |
| Direct effect (p-value) | | |
| β (95% CI) | -0.058 (-0.130,0.014) | -0.047 (-0.130,0.035) |
| p-value | 0.114 | 0.258 |

*Notes*, all analysis adjusted only for age, sex, pain intensity and injury severity.

For peer review only
Supplementary tables

Supplementary Table 3e
*Direct and indirect effects between predictors and functional outcome (GOS-E), adjusting for age, sex and injury severity*

| | Indirect effects | | | | Direct effects[a] |
|---|---|---|---|---|---|
| | B | (95% CI) | p-value | % of total effect mediated | p-value |
| **Mediation via Pain Self-Efficacy** | | | | | |
| Compensation status | -0.025 | -0.055,0.006 | 0.110 | 11.8% | 0.004 |
| Fault | -0.032 | -0.062,-0.001 | 0.041 | 19.4% | 0.032 |
| **Mediation via Pain Catastrophising** | | | | | |
| Fault | -0.026 | -0.054,0.002 | 0.065 | 16.4% | 0.023 |
| **Mediation via Perceived Injustice** | | | | | |
| Compensation status | -0.050 | -0.087,-0.013 | 0.009 | 23.5% | 0.006 |
| Fault | -0.084 | -0.125,-0.043 | <0.001 | 50.9% | 0.189 |

*Notes*, all analysis adjusted only for age, sex, pain intensity and injury severity.

Supplementary Table 3f
*Combined mediation effects for the relationship between GoS-E and compensation status/fault*

| | **Fault attribution** | **Compensation** |
|---|---|---|
| **Total indirect effect** | | |
| β (95% CI) | -0.089 (-0.133,-0.045) | -0.053 (-0.091,-0.016) |
| p-value | <0.001 | 0.006 |
| % of mediated | | |
| Total effect | 55.6% | 25.1% |
| Pain self-efficacy | 5.6% | 3.8% |
| Perceived injustice | 43.5% | 21.2% |
| Pain Catastrophising | 6.5% | n/a |
| **Direct effect (p-value)** | | |
| β (95% CI) | -0.071 (-0.192,0.051) | -0.159 (-0.278,-0.041) |
| p-value | 0.255 | 0.009 |

*Notes*, all analysis adjusted only for age, sex, pain intensity and injury severity.

For peer review only
Supplementary tables

**STROBE Statement—checklist of items that should be included in reports of observational studies**

**Manuscript title**: Compensation system exposure and fault attribution after traumatic injury: Associations with pain and disability
**Authors**: Giummarra et al.

| | Item No. | Recommendation | Page No. | Relevant text from manuscript |
|---|---|---|---|---|
| **Title and abstract** | 1 | (*a*) Indicate the study's design with a commonly used term in the title or the abstract | 1-2 | "prospective observational cohort study" |
| | | (*b*) Provide in the abstract an informative and balanced summary of what was done and what was found | 2 | |
| **Introduction** | | | | |
| Background/rationale | 2 | Explain the scientific background and rationale for the investigation being reported | 4-5 | |
| Objectives | 3 | State specific objectives, including any prespecified hypotheses | 5 | "We hypothesised that…" |
| **Methods** | | | | |
| Study design | 4 | Present key elements of study design early in the paper | 5+ | |
| Setting | 5 | Describe the setting, locations, and relevant dates, including periods of recruitment, exposure, follow-up, and data collection | 5-6 | |
| Participants | 6 | *Cohort study*—Give the eligibility criteria, and the sources and methods of selection of participants. Describe methods of follow-up<br>*Case-control study*—Give the eligibility criteria, and the sources and methods of case ascertainment and control selection. Give the rationale for the choice of cases and controls<br>*Cross-sectional study*—Give the eligibility criteria, and the sources and methods of selection of participants | 5-6 | |
| | | *Cohort study*—For matched studies, give matching criteria and number of exposed and unexposed<br>*Case-control study*—For matched studies, give matching criteria and the number of controls per case | n/a | |
| Variables | 7 | Clearly define all outcomes, exposures, predictors, potential confounders, and effect modifiers. Give diagnostic criteria, if applicable | 6-9 | |
| Data sources/ measurement | 8* | For each variable of interest, give sources of data and details of methods of assessment (measurement). Describe comparability of assessment methods if there is more than one group | 5-7 | |
| Bias | 9 | Describe any efforts to address potential sources of bias | 6 | "The recruitment strategy…" |
| Study size | 10 | Explain how the study size was arrived at | 8 | "the sample was sufficient…" |



For peer review only - http://bmjopen.bmj.com/site/about/guidelines.xhtml

| | | | | |
|---|---|---|---|---|
| Quantitative variables | 11 | Explain how quantitative variables were handled in the analyses. If applicable, describe which groupings were chosen and why | 8-9 | Data analytic approach |
| Statistical methods | 12 | (*a*) Describe all statistical methods, including those used to control for confounding | 8-9 | |
| | | (*b*) Describe any methods used to examine subgroups and interactions | 8-9 | |
| | | (*c*) Explain how missing data were addressed | 8 | "participants with missing data …" |
| | | (*d*) *Cohort study*—If applicable, explain how loss to follow-up was addressed<br>*Case-control study*—If applicable, explain how matching of cases and controls was addressed<br>*Cross-sectional study*—If applicable, describe analytical methods taking account of sampling strategy | n/a | |
| | | (*e*) Describe any sensitivity analyses | n/a | |
| **Results** | | | | |
| Participants | 13* | (a) Report numbers of individuals at each stage of study—eg numbers potentially eligible, examined for eligibility, confirmed eligible, included in the study, completing follow-up, and analysed | 9 | |
| | | (b) Give reasons for non-participation at each stage | 9 | |
| | | (c) Consider use of a flow diagram | -- | |
| Descriptive data | 14* | (a) Give characteristics of study participants (eg demographic, clinical, social) and information on exposures and potential confounders | 11<br>Table 1 | |
| | | (b) Indicate number of participants with missing data for each variable of interest | Tables/footnotes | N included each analysis is reported in Tables 2 & 3, and Table 4 footnotes |
| | | (c) *Cohort study*—Summarise follow-up time (eg, average and total amount) | n/a | |
| Outcome data | 15* | *Cohort study*—Report numbers of outcome events or summary measures over time | n/a | |
| | | *Case-control study*—Report numbers in each exposure category, or summary measures of exposure | n/a | |
| | | *Cross-sectional study*—Report numbers of outcome events or summary measures | n/a | |
| Main results | 16 | (*a*) Give unadjusted estimates and, if applicable, confounder-adjusted estimates and their precision (eg, 95% confidence interval). Make clear which confounders were adjusted for and why they were included | 12-15<br>Table 3<br>Figure 1-2 | |
| | | (*b*) Report category boundaries when continuous variables were categorized | Indicated in tables where relevant | |
| | | (*c*) If relevant, consider translating estimates of relative risk into absolute risk for a meaningful time period | n/a | |
| Other analyses | 17 | Report other analyses done—eg analyses of subgroups and interactions, and sensitivity analyses | n/a | |
| **Discussion** | | | | |
| Key results | 18 | Summarise key results with reference to study objectives | 17 | |
| Limitations | 19 | Discuss limitations of the study, taking into account sources of potential bias or imprecision. Discuss both direction and magnitude of any potential bias | 20 | |



For peer review only - http://bmjopen.bmj.com/site/about/guidelines.xhtml

| | | | |
|---|---|---|---|
| Interpretation | 20 | Give a cautious overall interpretation of results considering objectives, limitations, multiplicity of analyses, results from similar studies, and other relevant evidence | 17-20 |
| Generalisability | 21 | Discuss the generalisability (external validity) of the study results | Discussion (especially page 20) |
| **Other information** | | | |
| Funding | 22 | Give the source of funding and the role of the funders for the present study and, if applicable, for the original study on which the present article is based | 21 |

*Give information separately for cases and controls in case-control studies and, if applicable, for exposed and unexposed groups in cohort and cross-sectional studies.

**Note:** An Explanation and Elaboration article discusses each checklist item and gives methodological background and published examples of transparent reporting. The STROBE checklist is best used in conjunction with this article (freely available on the Web sites of PLoS Medicine at http://www.plosmedicine.org/, Annals of Internal Medicine at http://www.annals.org/, and Epidemiology at http://www.epidem.com/). Information on the STROBE Initiative is available at www.strobe-statement.org.

For peer review only

BMJ Open

# Associations between compensable injury, perceived fault and pain and disability one year after injury: A registry-based Australian cohort study

| | |
|---|---|
| Journal: | *BMJ Open* |
| Manuscript ID | bmjopen-2017-017350.R1 |
| Article Type: | Research |
| Date Submitted by the Author: | 04-Aug-2017 |
| Complete List of Authors: | Giummarra, Melita; Monash University, Department of Epidemiology and Preventive Medicine; Caulfield Pain Management and Research Centre,<br>Baker, Katharine; Monash University, School of Psychological Sciences<br>Ioannou, Liane; Monash University, School of Public Health & Preventive Medicine<br>Gwini, Stella; Monash University, Department of Epidemiology and Preventive Medicine<br>Gibson, Stephen; Caulfield Hospital, Caulfield Pain Management & Research Centre<br>Arnold , Carolyn ; Caulfield Hospital, Caulfield Pain Management & Research Centre<br>Ponsford, Jennie; Monash University, School of Psychological Sciences<br>Cameron, Peter; Monash University, Department of Epidemiology and Preventive Medicine |
| <b>Primary Subject Heading</b>: | Rehabilitation medicine |
| Secondary Subject Heading: | Epidemiology, Emergency medicine |
| Keywords: | Musculoskeletal Pain, Trauma, Trauma and Stressor Related Disorders, Insurance, Disability |
| | |

SCHOLARONE™
Manuscripts

**Title**: Associations between compensable injury, perceived fault and pain and disability one year after injury: A registry-based Australian cohort study

**Submission:** BMJ Open


**Authors**

1. Melita J. Giummarra [a,b,c*]
2. Katharine S. Baker [c,d*]
3. Liane Ioannou [d]
4. Stella M. Gwini [a]
5. Stephen J. Gibson [c]
6. Carolyn A. Arnold [c,f]
7. Jennie Ponsford [d,e]
8. Peter Cameron [a]

* Authors Melita Giummarra and Katharine Baker contributed equally to the paper, and are co-first author.

**Affiliations**

a) Department of Epidemiology and Preventive Medicine, School of Public Health & Preventive Medicine, Monash University, Melbourne, Australia
b) Institute for Safety, Compensation & Recovery Research, Monash University, Victoria, Australia
c) Caulfield Pain Management and Research Centre, Caulfield Hospital, Victoria, Australia
d) School of Psychological Sciences and Monash Institute of Cognitive and Clinical Neurosciences, Monash University, Faculty of Medicine, Nursing and Health Sciences, Clayton, Victoria, Australia
e) Monash-Epworth Rehabilitation Research Centre, Epworth Hospital, Richmond, Victoria, Australia
f) Academic Board of Anaesthesia & Perioperative Medicine, School of Medicine Nursing & Health Sciences, Monash University, Clayton, VIC, Australia

**Correspondence**

Dr Melita Giummarra
School of Public Health & Preventive Medicine
553 St Kilda Road
Monash University MELBOURNE VIC 3004
Ph: 61 3 9903 0365
Fax: 61 3 9905 3948
Email: melita.giummarra@monash.edu


For peer review only

## ABSTRACT

**Objectives.** Having a compensable injury increases the likelihood of having persistent pain after injury. Three quarters of patients report chronic pain after traumatic injury, which is disabling for one third of patients. It is important to understand why these patients report disabling pain, in order to develop targeted preventative interventions. This study examined the experience of pain and disability, and investigated their sequential interrelationships with, catastrophising, kinesiophobia and self-efficacy one year after compensable and non-compensable injury.

**Design.** Observational registry-based cohort study.

**Setting.** Metropolitan Trauma Service in Melbourne, Victoria, Australia.

**Participants.** Participants were recruited from the Victorian State Trauma Registry and Victorian Orthopaedic Trauma Outcomes Registry. 732 patients referred to the study, 82 could not be contacted or were ineligible, 217 declined, and 433 participated (66.6% response rate).

**Outcome measures.** The Brief Pain Inventory, Glasgow Outcome Scale, EuroQol Five Dimensions questionnaire, Pain Catastrophizing Scale, Pain Self-Efficacy Questionnaire, Injustice Experience Questionnaire and the Tampa Scale of Kinesiophobia.

**Methods.** Direct and indirect relationships (i.e., via psychological appraisals of pain/injury) between baseline characteristics (compensation, fault and injury characteristics), and pain severity, pain interference, health status and disability were examined with ordinal, linear and logistic regression, and mediation analyses.

**Results.** Injury severity, compensable injury and external fault attribution were consistently associated with moderate-severe pain, higher pain interference, poorer health status, and moderate-severe disability. The association between compensable injury, or external fault attribution, and disability and health outcomes was mediated via pain self-efficacy and perceived injustice.

**Conclusions.** Given that the association between compensable injury and disability and pain-related outcomes was attributable to lower self-efficacy and higher perceptions of injustice, interventions targeting the psychological impacts of pain and injury may be especially necessary to improve long-term injury outcomes.


**Key words:** Musculoskeletal Pain; Trauma; Trauma and Stressor Related Disorders; Insurance, Disability

For peer review only

## STRENGTH AND LIMITATIONS OF THIS STUDY

- Our understanding of the link between compensable injury and poor recovery has been limited by the varying nature of compensation system and systematic methodological factors, especially given that only those not at fault are eligible for compensation in many settings, and those with a poor recovery are more likely to lodge a claim.
- The regionalized trauma system in Victoria, Australia, facilitates systematic collaboration between the ambulance and retrieval services, trauma centers, and compensation systems, resulting in near complete identification of all compensable hospitalized injuries. This is, therefore, an ideal setting to investigate compensable injury outcomes.
- While the present sample was large and represented a range of injury severities, the findings should be taken in light of the biased sample socioeconomic characteristics, which had relatively higher socioeconomic status than the Victorian injury population. This cross-sectional observational cohort study identified theoretically based sequential associations between compensable injury (and fault attribution), psychological appraisals of pain and/or injury, and level of function and health status one year after injury.



For peer review only - http://bmjopen.bmj.com/site/about/guidelines.xhtml

## INTRODUCTION

Pain and injury are a leading contributors to global disease burden.[1] After traumatic injury,[2] disabling pain affects one in every 3-4 persons three years later,[3 4] making injury a significant cause of chronic pain in the community. Compensable injury, or the eligibility for and/or pursuit of an injury compensation claim, paradoxically leads to worse outcomes, including chronic and disabling pain.[5] This is despite the fact that compensation claimants are typically entitled to more benefits to support recovery, including healthcare and income replacement,[6-8] and some may receive lump sum payments depending on the setting.

Several factors may explain the "compensation effect". First, symptom exaggeration and malingering are thought to be present to varying degrees in up to thirty percent of injury claimants.[9] Moreover, those who seek compensation may selectively represent those who have a worse outcome (or those who are more likely to report poorer outcomes, e.g., seeking secondary gain[10]). It should also be noted that compensable injury (especially transport injury) typically involves more severe injury (e.g., multi-trauma). Altogether, these factors often result in misleading "reverse causality" explanations of the effect of compensation on recovery.[11] Nonetheless, even when studies account for injury characteristics those who had a compensable injury are still often found to have worse recovery.[12] Aside from methodological problems in the literature, key mechanisms through which compensable injury may result in poorer outcomes include the additive experience of stress from engaging with compensation systems (e.g., due to perceived lack of power),[13] or having to prove that another was at fault,[14] and the effects of several procedural factors on injustice and stress. Specific sources of procedural injustice include: (a) poor access to clear and timely information about compensation procedures or application outcomes, (b) perceived lack of empathy or engagement in interactions, and (c) dissatisfaction with decisions about individual entitlements.[15 16]

While compensable injury is consistently associated with poorer long-term injury outcomes, the mechanistic role of psychological appraisals of pain and/or the injury have rarely been examined.



For peer review only - http://bmjopen.bmj.com/site/about/guidelines.xhtml

A large body of work has demonstrated that pain catastrophizing (defined as the tendency towards having an exaggerated or excessive focus on negative aspects of pain and a lack of control over pain[17]) is associated with the persistence of pain and disability.[18 19] Fear of exacerbating pain or causing re-injury (i.e., kinesiophobia) and self-efficacy appraisals, which increase the likelihood of avoiding activity,[20 21] are also associated with worse disability,[22] and poorer quality of life in persons with persistent pain.[23-26] Moreover, persistent pain and disability after compensable injury are associated with negative appraisals of compensation-related experiences,[16] which may co-occur with maladaptive cognitive appraisals of pain and perceptions of injustice.[27-31] In fact, the *belief* that another was at fault, or to blame, is consistently associated with worse outcomes after compensable injury,[32 33] especially in settings where determinations of fault are central to eligibility for compensation.[34] Altogether, injustice appraisals and stress after injury may increase the likelihood of transitioning from acute to chronic pain due to their concurrent impacts on the person's behaviours and stress regulation systems, which may disrupt the capacity to process, regulate and cope with painful sensations.[35 36]

While many studies have shown that compensable injury is associated with greater likelihood of developing persistent pain,[5] whether persons who sustain a compensable injury have worse pain because they also have maladaptive appraisals of pain is not known. This observational registry-based cohort study examined the experience of pain, catastrophising, kinesiophobia and self-efficacy after compensable and non-compensable injury, and examined the association between these psychological factors in the experience of pain, disability and poor health status one year after injury. We hypothesised that those with a compensable injury, and those who perceived that another was at fault, would be more likely to report severe and disabling pain, and that these outcomes (i.e., pain and disability) would be mediated by lower self-efficacy, and higher pain catastrophizing, kinesiophobia, and perceived injustice.



For peer review only - http://bmjopen.bmj.com/site/about/guidelines.xhtml

For peer review only

## METHODS

### Participants and recruitment

Participants were recruited from the Victorian State Trauma Outcomes Registry (VSTR) and the Victorian Orthopaedic Trauma Outcomes Registry (VOTOR)[37-39] 12-months after admission to hospital for traumatic injury. Only English-speaking participants aged 18-70 were eligible to participate. Exclusion criteria were cognitive impairment as assessed qualitatively during trauma registry interview, participation in the registry via proxy representative, or high levels of distress. Distress was evaluated qualitatively by the registry interviewers, all of whom had worked in this role for several years, and was based on the participant's inability to complete the registry interview due to distress, or expressions of self-harm or suicidal ideation.

The VSTR and VOTOR registries are held in the Department of Epidemiology & Preventive Medicine, Monash University, and the same interviewers collect follow-up information for both registries. The registries comprise comprehensive details about patient demographics and injury and admission data, including trauma cause, mechanism and place, hospital admission, diagnoses and procedures. Injury and pain outcomes are assessed through telephone interviews at 6, 12 and 24 months following injury. The present study collected baseline and 12-month data from the registries, and administered additional questionnaires about pain, mental health and psychological factors related to the injury or pain (i.e., catastrophizing, kinesiophobia, self-efficacy, and perceived injustice) one year after injury.

Participants are included in VSTR if they meet major trauma criteria, defined as (a) admission to the intensive care unit for >24 hours and mechanically ventilated; (b) significant injury to two or more body regions (i.e., an Abbreviated Injury Scale (AIS, 2008 scoring criteria) score of >2 in two or more body regions) or a total Injury Severity Score (ISS) greater than 12; or (c) urgent surgery for intracranial, intrathoracic or intra-abdominal injury, or fixation of pelvic or spinal fractures. Patients are included in VOTOR if they had orthopaedic (bone or soft tissue) injuries not related to metastatic disease, and were admitted to hospital for > 24 hours. Patients are provided with information about



For peer review only

the registries before the first follow-up interview, and are given the opportunity to opt-off. Less than one percent of patients elect to be removed from VOTOR or VSTR.

The present strategy to recruit from both VSTR and VOTOR aimed to ensure that (a) the cohort comprised a range of injury severity; (b) potential sources of bias could be identified through comparison with other publications of these registry patients; and (c) reliance on patient recall or medical record review was minimised as injury and admission data were available from the trauma registries.

**Materials and Procedures**

The study was approved by Alfred Hospital (study: 290/13) and Monash University (study: CF13/3276 - 2013001633) human research ethics committees. Participants were invited into the present study by trauma registry staff at the conclusion of the 12-month registry interview if they were treated at The Alfred Hospital, one of the two major trauma services in Victoria, Australia. Participants were not informed of the specific study hypotheses, but that the study was examining which factors affect recovery from traumatic injury. Participants were reassured that their data would not be shared with any other parties. All participants gave informed written consent to participate in this study, and for the researchers to obtain data from the trauma registries. Participants then completed additional questionnaires either via telephone interview, online or in hardcopy.

*Demographics and pre-injury health*

Participant characteristics collected from the registries included sex, age at time of injury, education level and work status. Presence of comorbidities or other pre-existing health conditions at the time of hospital admission were determined using the International Statistical Classification of Diseases and Related Health Problems, Tenth Revision, Australian Modification (ICD-10-AM) diagnosis codes. Participants were also asked about other existing health conditions that might not have been captured at initial admission.

*Injury characteristics*



For peer review only - http://bmjopen.bmj.com/site/about/guidelines.xhtml

Injury data extracted from the trauma registries included Abbreviated Injury Scale (AIS) 2005 Update 2008 ,[40] Injury Severity Score (ISS; the sum of the three most severe AIS scores, squared, from different body regions),[41] length of stay in hospital (in days), and discharge destination (i.e., home or inpatient rehabilitation). In all cases, AIS scores were coded retrospectively by trained and experienced AIS coders either employed by the health service trauma registry or the Victorian State Trauma Registry. The maximum AIS score across body regions (i.e., head, neck, thorax, abdomen, spine, upper extremity, lower extremity, unspecified), and the number of body regions with an AIS score ≥2 (i.e., moderate to critical injuries) were used to reflect injury severity, as ISS has previously been shown to have little to no association with pain after injury when adjusting for other demographic and injury covariates.[39] Trauma place (i.e., transport, work, home or other), and whether or not the person felt they were at fault, were recorded.

The injury was defined as compensable if it was classified as such from the hospital records in VOTOR or VSTR, if the participant reported during our interviews that they had lodged a compensation claim (including Victims of Crime or public liability), or if the participant was eligible for compensation due to the setting and circumstances of their injury. That is, in Victoria, transport injury involving a motorized vehicle or a vehicle that operates on rails automatically qualifies for assistance from the Traffic Accident Commission (TAC), and injury while in the course of paid work is compensable by WorkSafe Victoria).

*Pain and functional outcomes (12 months)*

*The Brief Pain Inventory (BPI)* was used to quantify pain severity and interference of pain with various aspects of daily life on 11-point Numeric Rating Scales[42] (Cronbach α =.92 for pain severity subscale and .95 for pain interference subscale in the present cohort). Scores ≥4/10 were considered indicative of moderate-severe pain.[43 44]

Level of disability was measured using the extended version of the *Glasgow Outcome Scale (GOS-E)* [45] which classifies patient status into one of eight categories: death, vegetative state, lower severe disability, upper severe disability, lower moderate disability, upper moderate disability, lower



For peer review only - http://bmjopen.bmj.com/site/about/guidelines.xhtml

good recovery and upper good recovery. Disability status was determined from independence, work and leisure activity participation, and relationships with family and friends, and classified as "good" (i.e., lower-upper good recovery) or moderate-severe disability (i.e., vegetative state, lower severe disability, upper severe disability, lower moderate disability, upper moderate disability). The GOS-E has been shown to have good reliability and validity when using the structured interview format after head injury [45 46] and/or major trauma.[47]

The *EuroQol Five Dimensions questionnaire* (*EQ-5D*)[48] was used to measure general health outcomes relating to five domains: mobility, self-care, usual activities, pain or discomfort, and anxiety or depression. A summary score ranging from 0.00 to 1.00 was calculated using the UK indexed norms[49], where a score of 1.00 indicates the best health state, and 0.00 indicates the worst health outcome. The UK tariffs were used as these are most commonly applied across international studies,[50] including previous Australian registry-based studies.[51] The EQ-5D shows sound validity and sensitivity to injury outcomes.[50 51]

*Psychological mediators (12 months)*

The mediating effects of psychological characteristics related to pain were assessed by four measures: the Pain Catastrophizing Scale (PCS), Pain Self-Efficacy Questionnaire (PSEQ), Injustice Experience Questionnaire (IEQ) and the Tampa Scale of Kinesiophobia (TSK).

The *PCS* measured the tendency to have an exaggerated negative mindset in response to painful experiences[17]. It comprises 13 items, and respondents rated the degree to which they had certain thoughts and feelings when in pain (from 0 'not at all' to 4 'all the time'). All items were summed to create a total score (Cronbach α = .95 in the present sample).

The *PSEQ*[52] is a 10-item inventory assessing how confident a person was that they can cope with their pain and accomplish the activities of daily life despite their pain. Confidence in these abilities was rated on a scale from 0 'not at all confident' to 6 'completely confident', and items were summed to create a total score (Cronbach α = .96 in the present data).



For peer review only - http://bmjopen.bmj.com/site/about/guidelines.xhtml

The *IEQ*[53] is a 12-item questionnaire on which respondents indicate the frequency of certain thoughts from 0 'never' to 4 'all the time', reflecting blame or unfairness and irreparability of loss due to an injury, which are summed to create a total score (Cronbach α = .95 in the present data).

The *TSK*[54] is a 17-item self-report measure of kinesiophobia (i.e. fear of movement or fear of re-injury from movement). A total score was calculated by summing all responses after inverting items 4, 8, 12 and 16 (Cronbach α = .84 in the present data).

**Data analytic approach**

Data were analysed with Stata statistical software version 14.0 (StataCorp 2015; College Station, Texas). Significance was determined if α < 0.05, or if the 95% confidence did not include 1.00 (logistic and ordinal regression) or 0.00 (linear regression, mediation). Participants with missing data (<5.0% of cases across respective analyses) were excluded from the respective analysis in a list-wise manner. The data were summarised with descriptive statistics.

The design of the primary analyses is summarized in Figure 1. Ordinal regression examined ordinal variables (i.e., pain severity; 0 = no pain, <4 = low pain, >4 = moderate-severe pain),[43 44] linear regression examined continuous variables (i.e., pain interference and EQ-5D summary score), and logistic regression for binary variables (i.e., GOS-E; "good" recovery vs moderate-severe disability). Univariable regression models were fit to examine the relationship between each independent and dependent variable while controlling for age, sex, pain severity and injury severity (number of body regions with moderate-severe AIS score). Violation of the proportional odds assumption was assessed for ordinal models, and effects were reported in accordance with these assumptions.

-- Insert Figure 1 about here --

Mediation analyses examined the sequential relationship between the independent variables (compensation and fault status), via the mediating variables (i.e., self-efficacy, catastrophizing and perceived injustice), and the dependent variables (pain interference, health status and disability). The strength of indirect effects was only examined if the proposed mediator



For peer review only - http://bmjopen.bmj.com/site/about/guidelines.xhtml

was significantly associated with both the independent and dependent variables in preliminary linear and logistic regression analyses.[55] Mediated relationships were tested using the Sobel-Goodman mediation test with linear analyses for continuous factors (BPI interference, EQ-5D summary score) or logistic analyses for the categorical outcome (GOS-E), and bootstrapping with 500 case resamples. All mediation analyses adjusted for age, sex, injury severity (number of body regions with moderate-severe AIS score), and pain severity. The presence and strength of indirect (i.e., mediated) effects were determined from examination of the size of the coefficient, and the bootstrapped 95% confidence intervals such that effects were considered significant if the CI did not contain zero. The mediated effects were defined as "partial mediation" if the direct effect (path c') was smaller and of the same sign as the indirect effect but remained significant, or as "complete mediation" if the indirect effect equalled the total effect, and the direct effect (path c') was no longer significant [55]. Effect estimates were interpreted as *very small* (<.01), *small* ($\geq$.20), *moderate* ($\geq$.50), *large* ($\geq$.80), *very large* ($\geq$1.20) or *huge* ( $\geq$2.0).[56]

The sample (n = 433) was sufficiently powered for the univariate linear and logistic regression conducted (with adjustment for four covariates: age, sex, pain severity, injury severity (i.e., number of body regions with a moderate-severe AIS score)), and for detection of moderate bias-corrected bootstrapped indirect effects, which require a minimum sample of 377 and 400 cases, respectively.[57]

## RESULTS

### Cohort overview

All participants were admitted to hospital after traumatic injury from October 2012 to October 2014. A total of 732 patients were referred to the study during their 12-month VOTOR or VSTR registry interview. Seventy potential participants could not be contacted leaving 662 assessed for eligibility. Twelve participants were ineligible (two were deceased, seven were distressed, and



For peer review only - http://bmjopen.bmj.com/site/about/guidelines.xhtml

three were unwell), and 217 declined to participate resulting in a sample of 433 participants (66.6% response rate).

The average time from injury to follow-up was 13.50 months (SD = 1.60 months). The participants were predominately male (74.8%), average age at time of injury was 44.8 years (SD = 14.2), and the majority of participants had completed post-secondary school education (63.9%), which is slightly higher than the general Australian population whereby 61% of persons aged 15-64 have a post-school qualification [58]. Almost two thirds had a household income greater than AUD $60,000 per annum (60.9%) 12-months after injury, which is slightly higher than the national average household income of $52,000 [59]. One hundred and sixty nine participants had a compensable injury, including a transport-related injury (*n* = 141) or workplace injury (*n* = 28). See Table 1 for an overview of the cohort characteristics (n = 433).

Two hundred and sixty-seven (61.7%) patients were registered to both VSTR and VOTOR, 111 (25.6%) patients were in VOTOR only, and 55 patients were in VSTR only. Consistent with the registry inclusion criteria, participants recruited from VSTR had higher injury severity scores (ISS, and maximum AIS) than participants recruited from VOTOR only (ISS: Mean Difference = 9.39, 95% CI: 7.45 to 11.32; maximum AIS: Mean Difference = .89, 95% CI: .76 to 1.03). There was no difference between participants who were registered to VSTR and those who were registered to VOTOR only on reported pain severity (Mean difference = -0.016, 95% CI: -0.46 to 0.43), pain interference (Mean difference = 0.22, 95% CI: -0.29 to 0.74), pain catastrophizing (Mean difference = 1.01, 95% CI: -1.38 to 3.40), kinesiophobia (Mean difference = 0.21, 95% CI: -.1.57 to 1.99) or health status (Mean Difference = 0.013, 95% CI: -0.38 to 0.063) one year after injury. Participants recruited from VSTR had lower pain self-efficacy (Mean difference = -3.60, 95% CI: -6.71 to -0.50), and higher perceived injustice (Mean difference = 4.09, 95% CI: 1.15 to 7.03), and were also more likely to have moderate to severe disability one year after injury than those only registered to VOTOR (RR: 1.49, $p = 0.003$). This latter difference is expected given that permanent disability (e.g., due to cognitive, functional, social or psychological impairments) is more likely to arise after major trauma.



For peer review only - http://bmjopen.bmj.com/site/about/guidelines.xhtml

**Factors associated with pain severity**

Data on pain and pain-related outcomes are summarised in Table 2. At one year post-injury, the majority of participants reported pain of low severity (i.e., <4/10; n=258, 59.6%), 63 (14.5%) reported no pain at all, and 112 (25.9%) reported moderate-severe pain (i.e., ≥4/10). A relatively small proportion of participants had clinically significant scores across measures, including high pain interference (scores ≥4; *n* = 120, 27.8%), high catastrophizing (scores ≥ 30; *n* = 34, 7.9%), low self-efficacy (scores < 20; *n* = 26, 6.1%), high kinesiophobia (scores >40; *n* = 172, 39.9%), and high perceived injustice (scores ≥ 20; *n* = 159, 36.9%).

There was a modest correlation between age and pain severity ($r_s$ = .13, p<.006), and females were more likely to report moderate-severe pain than males (OR: 1.71; 95% CI: 1.06, 2.75). Participants with lower education (i.e., year 11 or below) were more likely to report moderate-severe pain (OR 2.80; 95% CI: 1.55, 5.05) than those with post-secondary education. Likewise, participants who were not employed prior to injury (RR 3.35, 95% CI 1.65, 6.81), or had not returned to work 12 months post injury (OR 2.95, 95% CI 1.69, 5.15), were more likely to report moderate-severe pain than those who were working before injury or had returned to work, respectively.

Relationships between baseline injury characteristics and pain severity one year post-injury are reported in Table 3. Participants were more likely to have pain if they had a more severe injury, such that for each additional body region with a moderate-critically severe injury, there was a 37% increase in the odds of having moderate-severe pain one year after injury. The likelihood of having moderate-severe pain was also associated with a longer hospital stay (4% increased odds of worse pain for each additional day), having a compensable injury (32% increased odds of pain), and attributing fault to another (46% increased odds of pain). However, place of injury (i.e. transport, work, home or elsewhere), compensation status and fault attribution were not related to pain severity when adjusting for injury and demographic characteristics.



For peer review only - http://bmjopen.bmj.com/site/about/guidelines.xhtml

Table 1

*Cohort characteristics*

| | Category | Total | | Compensable N = 169 | | Not Compensable N = 264 | |
|---|---|---|---|---|---|---|---|
| | | N | % | N | % | N | % |
| **Demographic characteristics** | | | | | | | |
| Sex | Male | 324 | 74.8 | 128 | 75.7 | 196 | 74.2 |
| | Female | 109 | 25.2 | 41 | 24.3 | 68 | 25.8 |
| Age at injury | 18-30 | 91 | 21.3 | 41 | 24.7 | 50 | 19.1 |
| | 31-40 | 70 | 16.4 | 23 | 13.9 | 47 | 17.9 |
| | 41-50 | 82 | 19.2 | 38 | 22.9 | 44 | 16.8 |
| | 51-60 | 122 | 28.5 | 42 | 25.3 | 80 | 30.5 |
| | 61+ | 63 | 14.7 | 22 | 13.3 | 41 | 15.6 |
| Presence of ≥1 comorbidity | None | 274 | 63.3 | 109 | 64.5 | 165 | 62.5 |
| | ≥1 | 159 | 36.7 | 60 | 35.5 | 99 | 37.5 |
| Highest education | Post-secondary education [a] | 272 | 64.5 | 102 | 63.4 | 170 | 65.1 |
| | Completed Year 12 | 64 | 15.2 | 27 | 16.8 | 37 | 14.2 |
| | Year 11 or less | 86 | 20.4 | 32 | 19.9 | 54 | 20.7 |
| Household income (p/a at 12-months after injury) | $20-40,000 | 98 | 23.6 | 40 | 26.3 | 58 | 22.1 |
| | $41-60,000 | 64 | 15.4 | 23 | 15.1 | 41 | 15.6 |
| | $61-80,000 | 67 | 16.1 | 30 | 19.7 | 37 | 14.1 |
| | $81-100,000 | 51 | 12.3 | 19 | 12.5 | 32 | 12.2 |
| | $100,000+ | 135 | 32.5 | 40 | 26.3 | 95 | 36.1 |
| **Work characteristics** | | | | | | | |
| Employment field | White Collar | 179 | 41.3 | 56 | 35.0 | 123 | 45.1 |
| | Blue Collar | 174 | 40.2 | 76 | 47.5 | 98 | 35.9 |
| | Not Working/Studying | 80 | 18.4 | 28 | 17.5 | 52 | 19.1 |
| **Injury characteristics** | | | | | | | |
| Moderate-Critical Injury[b] | 1. Head | 120 | 27.7 | 59 | 34.9 | 61 | 23.1 |
| | 2. Face | 82 | 18.9 | 42 | 24.9 | 40 | 15.2 |
| | 3. Neck | 12 | 2.8 | 8 | 4.7 | 4 | 1.5 |
| | 4. Thorax | 146 | 33.7 | 90 | 53.3 | 56 | 21.2 |
| | 5. Abdomen | 50 | 11.5 | 39 | 23.1 | 11 | 4.2 |
| | 6. Spine | 151 | 34.9 | 65 | 38.5 | 86 | 32.6 |
| | 7. Upper Extremity | 165 | 38.1 | 77 | 45.6 | 88 | 33.3 |
| | 8. Lower Extremity | 218 | 50.3 | 100 | 59.2 | 118 | 44.7 |
| | 9. Unspecified | 35 | 8.1 | 16 | 9.5 | 19 | 7.2 |
| Discharge destination | Home | 304 | 70.2 | 92 | 54.4 | 212.0 | 80.3 |
| | Rehabilitation | 129 | 29.8 | 77 | 45.6 | 52.0 | 19.7 |



For peer review only - http://bmjopen.bmj.com/site/about/guidelines.xhtml

[a] Post-secondary education included post-secondary school certificate, diploma, bachelor or post-graduate degree; [b] Body region with severe injury with an AIS severity score of 2-5, and multiple body regions could be affected for each participant.

Table 2

*Pain and pain-related characteristics in compensable and non-compensable participants*

| | Measure | Statistic | Compensable N = 160 | | Not Compensable N = 273 | | P | Effect size |
|---|---|---|---|---|---|---|---|---|
| Pain Severity | BPI | M(SD) | 2.94 | (2.19) | 2.30 | (1.94) | .002 | .31 |
| Pain interference | BPI | M(SD) | 3.39 | (2.78) | 2.16 | (2.28) | <.001 | .48 |
| Pain catastrophising | PCS | Md(IQR) | 8.00 | (16.00) | 4.00 | (13.00) | <.001* | .17 |
| Pain self-efficacy | PSEQ | M(SD) | 41.41 | (15.43) | 47.78 | (13.14) | <.001 | .44 |
| Kinesiophobia | TSK | M(SD) | 38.45 | (8.39) | 36.30 | (7.99) | .008 | .26 |
| Perceived injustice | IEQ | M(SD) | 20.52 | (14.61) | 13.73 | (12.40) | <.001 | .50 |

*Notes*: Statistics were all independent samples t-tests, and Cohen's D effect sizes, except for pain catrastophizing, which was examined with a non-parametric Mann Whitney U tests (and effect size calculation of z/√N).

*Abbreviations*: BPI = Brief Pain Inventory; PCS = Pain Catastrophizing Scale; PSEQ = Pain Self-Efficacy Questionnaire; TSK = Tampa Scale of Kinesiophobia; IEQ = Injustice Experience Questionnaire; M = Mean; SD = Standard Deviation; Md = Median; IQR = the range between the 25th and 75th percentile.

For peer review only

Table 3

*Relationship between injury characteristics and pain severity (ordinal regression)*

| Characteristics | | No Pain N = 63 (14.5%) | Low Pain N = 258 (59.6%) | Mod-Severe Pain N = 112 (25.9%) | OR | $OR^{adj}$ (95% CI) |
|---|---|---|---|---|---|---|
| **Injury severity** | | | | | | |
| AIS count+ | M (SD) | 1.51 (0.82) | 1.75 (1.07) | 2.13 (1.33) | 1.38 | 1.37 (1.15, 1.62)* |
| **Hospital Stay (continuous)++** | | | | | | |
| None vs any pain | M (SD) | 6.49 (6.36) | 6.72 (8.13) | | 1.00 | 0.98 (0.95, 1.02) |
| None/low vs mod/severe pain | M (SD) | 5.69 (5.99) | | 9.53 (11.31) | 1.05* | 1.04 (1.01, 1.07)* |
| **Injury place** | | | | | | |
| At home | N (%) | 14 (22.2) | 45 (17.4) | 18 (16.1) | Ref | Ref |
| Traffic/Road | N (%) | 23 (36.5) | 96 (37.2) | 54 (48.2) | 1.52 | 1.38 (0.75, 2.52) |
| Workplace | N (%) | 4 (6.4) | 25 (9.7) | 16 (14.3) | 1.98 | 1.99 (0.93, 4.26) |
| Other | N (%) | 22 (34.9) | 92 (35.7) | 24 (21.4) | 0.88 | 1.11 (0.60, 2.06) |
| **Compensation status** | | | | | | |
| None | N (%) | 41 (65.1) | 169 (65.5) | 55 (49.1) | Ref | Ref |
| TAC/Worksafe | N (%) | 22 (34.9) | 89 (34.5) | 57 (50.9) | 1.68* | 1.32 (0.84, 2.07) |
| **Fault** | | | | | | |
| At fault | N (%) | 36 (57.1) | 133 (52.0) | 46 (41.8) | Ref | Ref |
| Not at fault | N (%) | 27 (42.9) | 123 (48.0) | 64 (58.2) | 1.50* | 1.46 (0.99, 2.15) |

*Notes*: Significant relationships are with an asterix (*). $OR^{adj}$ have adjusted for age, sex and education. Analysis of Hospital stay, injury place, compensation, fault, and work status also controlled for injury severity (number of body regions with moderate-severe AIS score).

+ AIS count = the number of mod-critical injured body regions,

++ The proportional odds assumption was not met for length of hospital stay, so ORs are reported here for each ordinal comparison.



For peer review only - http://bmjopen.bmj.com/site/about/guidelines.xhtml

**Factors associated with  psychological variables (*a* paths)**

Figure 2 shows associations between baseline injury characteristics and psychological functioning in relation to pain at 12 months (adjusting for age, sex, pain severity and injury severity). Catastrophizing, self-efficacy and perceived injustice were all worse in those who were discharged to inpatient rehabilitation following their injury, and in those who attributed fault to another. Self-efficacy was lower in participants who had a compensable injury or a longer hospital stay. Perceived injustice was worst in participants with transport or work-related injuries compared to those with an injury at home or elsewhere, in participants with  compensable injury, and with longer hospital stay. Kinesiophobia was not related to any injury characteristics.

-- Insert Figure 2 about here --

**Factors associated with poor functional recovery (direct effects and Path *b*)**

Examination of the direct effects of injury characteristics and psychological responses to the pain or injury on pain interference were examined only in participants who reported some pain 12-months after injury (*n* = 370), see Figure 3. Most participants reported low levels of pain interference (<4/10; n=249, 67.5%), and the remainder (n=120, 32.3%) reported moderate to severe pain interference (i.e. ≥4/10), with average pain interference of 3.04 (SD = 2.49) in participants reporting some pain.

The relationships between injury characteristics and psychological responses to the pain or injury and the EQ-5D and GOS-E were examined in all participants. The averageEQ-5D summary scores was .80 (SD = .23), indicating moderate-to-good health status in the majority of participants. According to the GOS-E, 210 (48.5%) participants had “good” functional recovery, 216 (49.9%) had moderate disability and seven (1.6%) patients had severe disability. Given the small number of patients who had severe disability, the moderate and severe disability groups were combined for all analyses.

Figure 3 shows the relationship between baseline characteristics and the functional and health-related dependent variables. Participants had poorer health or function across all three



For peer review only - http://bmjopen.bmj.com/site/about/guidelines.xhtml

measures (BPI Interference; EQ-5D; GOS-E) if they sustained a compensable injury, attributed fault to another or required inpatient rehabilitation (see Supplementary Materials for specific ORs and CIs). At one year post-injury, disability (GOS-E) was more likely in those who were *employed* prior to injury, whereas pain interference and health status were worse in those who were *unemployed* prior to injury. Pre-existing medical conditions were not associated with any dependent variable; however, it should be noted that the sample generally had good health prior to injury, with only 132 patients (35.7%) reporting one or more comorbidities at the time of the injury, and the cohort had an average rating of pre-injury health of 89.16 (SD=10.76) out of 100, where 100 indicates "best imaginable health state".

All psychological variables (self-efficacy, kinesiophobia, catastrophizing, and perceived injustice) were predictive of higher pain interference, lower health status, and disability outcome after controlling for demographics, pain severity and injury severity (Table 4).

-- Insert Figure 3 about here --

Table 4

*Association between mediators and pain interference, health status (EQ-5D summary score), and GOS-E recovery [Path b]*

| | Pain Interference | | EQ-5D Summary Score | | GOS-E Functional Outcome | |
|---|---|---|---|---|---|---|
| **Mediators** | **B** | **(95% CI)** | **β** | **(95% CI)** | **OR** | **(95% CI)** |
| Pain Severity | 1.02 | 0.95,1.10 | -0.065 | -0.077,-0.053 | 0.59 | 0.52,0.68 |
| Pain Self-Efficacy | -0.06 | -0.08,-0.04 | 0.005 | 0.003,0.007 | 1.04 | 1.01,1.06 |
| Kinesiophobia | 0.07 | 0.04,0.09 | -0.004 | -0.007,-0.001 | 0.95 | 0.91,0.98 |
| Catastrophising | 0.08 | 0.06,0.10 | -0.007 | -0.010,-0.004 | 0.95 | 0.91,0.98 |
| Perceived Injustice | 0.06 | 0.04,0.07 | -0.004 | -0.006,-0.003 | 0.94 | 0.92,0.96 |

*Notes*: all analysis adjusted only for age, sex, pain severity and injury severity (number of body regions with moderate-severe AIS score). The sample for pain interference regression only comprised participants reporting a pain intensity>0; N=370). Pain interference and EQ-5D summary score were analyzed with linear regression, GOS-E was analyzed with logistic regression (comparing "good" recovery vs moderate to severe disability, where higher odds indicate increased likelihood of the good recovery).



For peer review only - http://bmjopen.bmj.com/site/about/guidelines.xhtml

**Indirect effects on functional outcomes (ab path; Path c')**

As kinesiophobia was not associated with compensation or fault, and catastrophizing was not associated with compensation, these variables were not included as potential mediators in the respective analyses. The effect estimates, and bootstrapped 95% CIs, of the indirect and direct effects are shown in Table 5.

*Pain interference*

There was partial mediation between compensation and pain interference via pain self-efficacy and perceived injustice, and between fault and pain interference via pain self-efficacy, perceived injustice and catastrophizing. The size of the direct and indirect effects were small to moderate, with the combined effect of the mediators explaining 59.3% and 48.7% of the total variance in the association between compensation, or fault attribution, and pain interference, respectively.

*Health*

There was complete mediation of the relationship between compensation and health status via self-efficacy and perceived injustice. There was also complete mediation of the relationship between fault and health status, and disability, via perceived injustice, but only partial mediation between fault and health status via self-efficacy and catastrophizing. While the magnitude of both the direct and indirect effects between compensation and fault on health status would be considered very small, with all estimates being less than - 0.04, it should be noted that 54.0% and 50.1%, respectively, of the total variance in the association was indirect via self-efficacy, perceived injustice and catastrophizing.

*Disability*

There was partial mediation between compensation and disability via perceived injustice (of moderate effect size), but no mediation via self-efficacy. There was complete mediation between fault and disability via perceived injustice (of small effect size), partial mediation via self-efficacy (moderate effect size), and no mediation via catastrophizing. The combined indirect effects, via the



For peer review only - http://bmjopen.bmj.com/site/about/guidelines.xhtml

For peer review only

mediators, explained 55.6% and 25.1% of the total variance in the association between compensation, or fault attribution, and disability, respectively.

For peer review only



For peer review only - http://bmjopen.bmj.com/site/about/guidelines.xhtml

Table 5

*Direct (path c') and indirect (mediated, path ab) effects, with bootstrapped 95% confidence interval (CI), between independent variables (compensation; fault) and functional or health status (BPI Interference; EQ-5D; GOS-E), mediated by psychological characteristics (pain self-efficacy; perceived injustice; pain catastrophizing)*

| | **Pain interference (BPI)** | | **Health (EQ-5D)** | | **Disability (GOS-E)** | |
|---|---|---|---|---|---|---|
| | Path *ab* Indirect effect | Path *c'* Direct effect | Indirect (mediated) effect | Direct effect* | Indirect (mediated) effect | Direct effect* |
| | *B* (95% CI) | *B* (95% CI) | *B* (95% CI) | *B* (95% CI) | *B* (95% CI) | *B* (95% CI) |
| **Pain Self-efficacy** | | | | | | |
| Compensation | 0.24 (0.07,0.41) | 0.41 (0.08, 0.75) | -0.02 (-0.29,-0.002) | -0.03 (-0.07, 0.009) | -0.25 (-0.06,0.01) | -0.19 (-0.31, -0.07) |
| Fault | 0.25 (0.09,0.41) | 0.42 (0.13, 0.72) | -0.02 (-0.35,-0.004) | -0.04 (-0.08, -0.007) | -0.32 (-0.06,-0.001) | -0.13 (-0.25, -0.01) |
| **Perceived injustice** | | | | | | |
| Compensation | 0.25 (0.10,0.40) | 0.39 (0.08, 0.70) | -0.02 (-0.03,-0.01) | -0.03 (-0.07, 0.01) | -0.05 ( -0.09,-0.01) | -0.16 (-0.28, -0.05) |
| Fault | 0.34 (0.19,0.48) | 0.33 (0.02, 0.63) | -0.03 (-0.04,-0.02) | -0.03 (-0.06, 0.004) | -0.08 (-0.13,-0.04) | -0.08 (-0.20, 0.04) |
| **Catastrophizing** | | | | | | |
| Fault | 0.17 (0.03,0.32) | 0.49 (0.18, 0.77) | -0.01 (-0.03,-0.002) | -0.04 (-0.07, -0.01) | -0.03 (-0.05,0.002) | -0.13 (-0.25, -0.01) |
| **Combined mediated effects** | | | | | | |
| Compensation | 0.07 (0.03,0.10) | 0.06 (0.006, 0.13) | -0.05 (-0.08,-0.02) | -0.05 (-0.13, 0.03) | -0.05 (-0.09,-0.02) | -0.16 (-0.27, -0.05) |
| Fault | 0.08 (0.04,0.12) | 0.05 (-0.0005, 0.11) | -0.07 (-0.10,-0.03) | -0.06 (-0.13, 0.01) | -0.09 (-0.13,-0.05) | -0.07 (-0.19, 0.05) |

*Note*: Analyses were univariate, adjusting for age, sex, pain severity and injury severity (number of body regions with moderate-severe AIS score).

* Direct effect while accounting for the effects of the mediator, and change in the magnitude of the direct effect should refer to the effects shown in Figure 2. Effects are considered statistically significant if the 95% confidence interval does not contain zero.



For peer review only - http://bmjopen.bmj.com/site/about/guidelines.xhtml

## DISCUSSION

This study demonstrates that characteristics at the time of injury, especially compensable injury and attributing fault to another, were consistently associated with poorer health and level of function one year after injury, including pain-related disability. These associations were observed both before and after controlling for injury severity and demographic factors that were also associated with worse outcomes. A notable exception was that pain *severity* one year after compensable injury appeared to be most strongly associated with injury severity. So while this study has replicated the so called "compensation effect",[5 6 60] we suggest that the mechanism of injury (i.e., transport crashes) for the majority of the compensable cases in this study may have increased the likelihood of having moderate-severe pain. This may arise due to the fact that transport-related injuries tend to arise from high energy collisions, and result in complex multi-trauma. Despite the clear association between injury severity and pain, we have shown for the first time that lower self-efficacy and higher perceptions of injustice after compensable injury mediated the degree to which pain impacted on a range of daily activities, as well as health and disability outcomes at one year after injury.

The total effect of fault perceptions on disability was found to be indirect via perceived injustice. While each of these factors no doubt covary after injury, and the sequential relationships could really be examined in varying combinations (e.g., disability after injury also leads to perceptions of injustice), these findings are consistent with the frequent finding that external attributions of fault lead to a range of poor health outcomes.[51 61] Here we show that when adjusting for injury severity, both attributions of fault and global perceptions of injustice are associated with reduced likelihood of having a good functional outcome. In other contexts, perceptions of injustice have been shown to have real and significant effects on rehabilitation outcomes, highlighting that disability is associated with a range of factors beyond the physical and functional limitations imposed by the injury. In fact, the harmful effects of perceived injustice have been shown to begin relatively early in the disability trajectory,[62] affect the quality of working relationship with health



For peer review only - http://bmjopen.bmj.com/site/about/guidelines.xhtml

professionals,[63] promote behaviours that are not conducive to recovery,[32] and lead to an inflexible focus on justice violations that may ultimately impede recovery.[33] In the worst case scenario, injustice appraisals may even lead to chronic embitterment and a range of long term mental health impacts, including depression and suicidal ideation.[64] Clearly, therefore, it is important to address injustice perceptions after injury, promote rehabilitation gains as early as possible, and attenuate any extrinsic contributors that exacerbate injustice perceptions (i.e., procedural injustice).

This study demonstrated an association between compensable injury and worse disability and health outcomes (i.e. in relation to mobility, self-care, activity participation, pain, and anxiety/depression), which were to varying degrees attributable to the experience of lower self-efficacy and higher injustice perceptions. These findings suggest that patients who had a compensable injury, compared with those who sustained a non-compensable injury, were more likely to lack confidence in participating in activities of daily living because of persistent pain. Further to the impacts of injustice perceptions on behaviour, described above, low self-efficacy is known to increase the likelihood of adopting maladaptive behaviours and thoughts, such as pain avoidance and reduced participation in work, social and physical activities.[65] While self-efficacy did not mediate the relationship between compensable injury and disability in this cohort one year after injury, given that compensable injury leads to low pain self-efficacy it may be that it will lead to greater disability beyond this time-frame.[23] Promoting self-efficacy, especially after compensable injury, is therefore a high priority for optimising health status and reducing pain interference one year after injury.[25]

While pain catastrophizing was not worse after compensable injury, it did show a small association with pain interference, and it partially mediated the relationship between fault and pain interference, and health status. The effects via catastrophizing were very small across all analyses, which is most likely due to the fact that catastrophizing characteristics were low in this cohort (i.e., more than ninety percent of participants had catastrophizing scores below the clinical threshold). However, it should be noted that this is twice as high as the proportion of patients with clinically elevated catastrophizing 6-months after musculoskeletal injury.[66] In the present study, only a



For peer review only - http://bmjopen.bmj.com/site/about/guidelines.xhtml

quarter of the sample had moderate to severe pain, but just over half of the sample had moderate to severe disability. Therefore, we suggest that the unjust impacts of the injury, as measured by the IEQ,[31 67] may have been more pertinent in this cohort than pain-related catastrophising, as measured by the PCS.

Although kinesiophobia was associated with worse functional outcomes, it was not associated with any injury characteristics, including compensation or fault attributions. Evidently fear of re-injury, or exacerbating pain, is not closely associated with the severity of the initial injury. Rather, we speculate that emerging functional and psychological impacts of the injury, together with enduring personality traits, may play a greater role in kinesiophobia than injury severity.

**Clinical implications**

It is clear that some injury and demographic characteristics increase the risk of persistent pain and disability after injury. There remains a pressing need now to develop and test effective psychosocial and medical interventions during the first year after injury to further improve long-term outcomes. Moreover, efforts should focus on modifying compensation procedures that exacerbate pain or psychological outcomes, and supporting recovery in those who believe that another was at fault, whether or not that belief is accurate.[51] Given that self-efficacy and perceived injustice showed important direct and indirect associations with function and health, further investigation is needed to understand whether these appraisals can be modified when specifically targeted in interventions.

At this stage, research on early interventions for the prevention of pain, disability, and injustice beliefs after injury is sparse.[33 68] Interventions delivered in the acute or sub-acute stage after injury that have been shown to have positive effects on self-efficacy typically comprise education,[69] and work towards building "mastery" of activities that had become difficult because of pain. These interventions use behavioural achievements as a catalyst for positive change (i.e. improved functional outcomes), which is more powerful than verbal encouragement alone.[25] Disability and perceptions of injustice are clearly bidirectionally associated.[33 53] Interventions targeting either injustice beliefs or functional restoration appear to elicit positive effects on the



For peer review only - http://bmjopen.bmj.com/site/about/guidelines.xhtml

other.[70] New interventions could be developed and trialled to modulate injustice beliefs directly, especially for persons with injuries that result in permanent disability (e.g., after spinal cord injury or brain injury). While injured persons may have very valid grounds for their beliefs, they may nonetheless benefit from therapies that enhance emotion control, acceptance [71] or forgiveness [72]. Ultimately, when designing any intervention to target complex psychological, pain and disability outcomes after injury it is important to bear in mind that feelings of injustice frequently extend far beyond the person at fault for causing the injury, and may be directed toward the compensation system, employers, health care providers, lawyers, and society as a whole.[62 73 74] It is therefore important that therapists and policy makers take a whole of person, and whole of system, approach to supporting injury recovery. Finally, procedures involved in claiming compensation, such as receiving timely and sufficient information, or having empathic interactions with claims staff, were not evaluated in this study, but should be evaluated to ensure that these procedures are not causing secondary harm.[15 73] Indeed, compensation systems are in a valuable position whereby they can optimise their systems and client relationships to bolster client self-efficacy.

**Strengths, limitations and future directions**

The strengths and limitations of the present study should be considered when applying these findings to the trauma population. First, in the State of Victoria, all persons who are injured in transport (i.e., involving a motorized or rail-operated vehicle) or workplace injuries are eligible for compensation, regardless of their role in the injury incident (i.e., this is a “no fault” system). When hospitalized, most cases will almost automatically have a claim number generated, in line with improved procedures for claim lodgment in cases that meet medical excess criteria for a claim. This setting is ideal for the examination of outcomes related to fault attributions, and compensable injury. That said, it should be noted that the present cohort, relative to the general population and trauma population studies in Victoria, had a relatively higher socioeconomic status (i.e., slightly higher proportion of patients with post-secondary education[58] and annual income [59] than the national average).



For peer review only - http://bmjopen.bmj.com/site/about/guidelines.xhtml

All of the mediating and dependent variables were measured one-year post-injury. Although the present analyses were theoretically driven (i.e., given that *beliefs* about pain, capacity to participate in activity, are predictive of actual behavior and disability), these characteristics are known to frequently covary. Therefore causal associations between psychological appraisals of pain or the injury (i.e., catastrophizing, self-efficacy, kinesiophobia and perceived injustice) and pain interference, health status and disability are not assumed, and further research is required to confirm these associations, and their potential for change through intervention. Despite the cross-sectional nature of the study, our findings highlight that self-efficacy and perceived injustice are powerful indicators of poor injury outcomes alongside (or perhaps more so than) injury severity, and should be considered during injury rehabilitation.

In conclusion, the present findings suggest that pain is more likely after compensable injury, but largely because these injuries are more severe and complex. When accounting for injury severity, however, compensable injury was nonetheless found to lead to worse self-efficacy and health status, and higher perceived injustice, pain-related disability (pain interference), and disability. As perceived injustice and low self-efficacy played a key role in pain, health and disability after compensable injury, these characteristics warrant further investigation as a risk factor for pain and disability, and as targets for intervention.



For peer review only - http://bmjopen.bmj.com/site/about/guidelines.xhtml

For peer review only

## FOOTNOTES

**Acknowledgements:** We acknowledge the contribution of Nellie Georgiou-Karistianis and Paul Jennings in the general design of the study, Melissa Hart and Mimi Morgan for participant recruitment, Susan McLellan for data extraction and injury coding, Amy Allen, Jessica Frisina and Samantha Finan for data collection.

**Contributors:** Each co-author contributed significantly to the work described and commented on the manuscript. MJG designed and supervised the conduct of the study, contributed to data preparation and analysis, discussed the results, and co-wrote the manuscript with guidance and feedback from the co-authors. KSB assisted with patient recruitment and data collection, prepared data for analysis, and contributed to interpretation and writing of the manuscript. LI assisted with patient recruitment and data collection, preparation of data for analysis, interpretation and writing of the manuscript. SMG assisted with data analysis, interpretation of findings, and writing and editing of the manuscript. SJG, CAA, JP and PC assisted in designing the project and analysis, interpretation of the findings, and review of the manuscript.

**Funding:** This research was supported by an Australian Research Council (ARC) Linkage Grant [LP120200033] in collaboration with the Victorian Transport Accident Commission (TAC), and a Platform Access Grant from Monash University [PAG15-0026]. MJG was supported by an NHMRC early career fellowship [APP1036124], and PC was supported by a NHMRC fellowship [APP545926]. The Victorian Orthopaedic Trauma Outcomes Registry (VOTOR) is funded by the Transport Accident Commission via the Institute for Safety, Compensation and Recovery Research (ISCRR). The Victorian State Trauma Registry (VSTR) is a Department of Health, State Government of Victoria and Transport Accident Commission funded project. The Victorian State Trauma Outcome Registry and Monitoring (VSTORM) group is thanked for the provision of VSTR data. The funders were consulted throughout the design of the study, but played no role in data collection and analysis, the decision of how and what to publish, or the preparation of this manuscript.

**Competing interests:** None declared.

**Ethics approval:** Alfred Hospital (study: 290/13) and Monash University (study: CF13/3276 – 2013001633) human research ethics committees.

**Data sharing statement:** The Victorian State Trauma Registry (VSTR) and the Victorian Orthopaedic Trauma Outcomes Registry (VOTOR) have strict data/publication policies and guidelines to protect against unauthorized use or misinterpretation of the data. Data is only available upon request from the corresponding author (MJG).

For peer review only

**REFERENCES**


1. Murray CJ, Vos T, Lozano R, et al. Disability-adjusted life years (DALYs) for 291 diseases and injuries in 21 regions, 1990–2010: a systematic analysis for the Global Burden of Disease Study 2010. *The Lancet* 2012;380(9859):2197-223. doi: 10.1016/S0140-6736(12)61689-4
2. O'Donnell ML, Varker T, Holmes AC, et al. Disability after injury: the cumulative burden of physical and mental health. *The Journal of clinical psychiatry* 2013;74(2):e137-43. doi: 10.4088/JCP.12m08011
3. Holmes AC, Williamson O, Hogg M, et al. Determinants of chronic pain 3 years after moderate or serious injury. *Pain medicine* 2013;14(3):336-44.
4. Holmes AC, Williamson O, Hogg M, et al. Predictors of pain 12 months after serious injury. *Pain medicine* 2010;11(11):1599-611.
5. Giummarra MJ, Ioannou L, Ponsford J, et al. Chronic pain following motor vehicle collision: A systematic review of outcomes associated with seeking or receiving compensation. *The Clinical journal of pain* 2016;in press, DOI: 10.1097/AJP.0000000000000342
6. Rasmussen C, Leboeuf-Yde C, Hestbaek L, et al. Poor outcome in patients with spine-related leg or arm pain who are involved in compensation claims: a prospective study of patients in the secondary care sector. *Scandinavian journal of rheumatology* 2008;37(6):462-8. doi: 10.1080/03009740802241709
7. Harris I, Young J, Jalaludin BB, et al. Predictors of neck pain after motor vehicle collisions: A prospective survey. *Journal of Orthopaedic Surgery* 2011;19(3):317-21.
8. Cassidy JD, Carroll LJ, Cote P, et al. Effect of eliminating compensation for pain and suffering on the outcome of insurance claims for whiplash injury. *New England Journal of Medicine* 2000;342(16):1179-86.
9. Bass C, Halligan P. Factitious disorders and malingering: challenges for clinical assessment and management. *Lancet* 2014;383(9926):1422-32. doi: 10.1016/s0140-6736(13)62186-8
10. Shuman DW. The psychology of compensation in tort law. *Kansas Law Rev* 1994;43:39–77.
11. Spearing NM, Connelly LB, Nghiem HS, et al. Research on injury compensation and health outcomes: ignoring the problem of reverse causality led to a biased conclusion. *Journal of clinical epidemiology* 2012;65(11):1219-26. doi: 10.1016/j.jclinepi.2012.05.012
12. Gabbe BJ, Simpson PM, Harrison JE, et al. Return to Work and Functional Outcomes After Major Trauma: Who Recovers, When, and How Well? *Annals of surgery* 2016;263(4):623-32. doi: 10.1097/sla.0000000000001564 [published Online First: 2016/01/19]
13. O'Donnell ML, Grant G, Alkemade N, et al. Compensation seeking and disability after injury: the role of compensation-related stress and mental health. *The Journal of clinical psychiatry* 2015;76(8):e1000-5. doi: 10.4088/JCP.14m09211
14. Elbers NA, Collie A, Hogg-Johnson S, et al. Differences in perceived fairness and health outcomes in two injury compensation systems: a comparative study. *BMC public health* 2016;16:658. doi: 10.1186/s12889-016-3331-3
15. Kilgour E, Kosny A, Akkermans A, et al. Procedural Justice and the Use of Independent Medical Evaluations in Workers' Compensation. *Psychological Injury and Law* 2015;8(2):153-68. doi: 10.1007/s12207-015-9222-6




For peer review only - http://bmjopen.bmj.com/site/about/guidelines.xhtml

16. Ioannou L, Braaf S, Cameron P, et al. Compensation System Experience at 12 Months After Road or Workplace Injury in Victoria, Australia. *Psychological Injury and Law* 2016;9(4):376-89. doi: 10.1007/s12207-016-9275-1
17. Sullivan MJL, Bishop SR, Pivik J. The pain catastrophizing scale: Development and validation. *Psychological Assessment* 1995;7(4):524-32. doi: 10.1037/1040-3590.7.4.524
18. Lindal E, Breivik H. Fear and catastrophizing thoughts aggravate risks of chronic pain after a fracture. *Scandinavian Journal of Pain* 2010;1(1):7. doi: 10.1016/j.sjpain.2009.09.007
19. Gopinath B, Jagnoor J, Nicholas M, et al. Presence and predictors of persistent pain among persons who sustained an injury in a road traffic crash. *European journal of pain* 2015;19(8):1111-8. doi: 10.1002/ejp.634
20. Vlaeyen JW, Linton SJ. Fear-avoidance and its consequences in chronic musculoskeletal pain: A state of the art. *Pain* 2000;85(3):317-32.
21. Vlaeyen JW, Linton SJ. Fear-avoidance model of chronic musculoskeletal pain: 12 years on. *Pain* 2012;153(6):1144-7. doi: 10.1016/j.pain.2011.12.009
22. Picavet HSJ. Pain Catastrophizing and Kinesiophobia: Predictors of Chronic Low Back Pain. *American Journal of Epidemiology* 2002;156(11):1028-34. doi: 10.1093/aje/kwf136
23. Arnstein P, Caudill M, Mandle CL, et al. Self-efficacy as a mediator of the relationship between pain intensity, disability and depression in chronic pain patients. *Pain* 1999;80(3):483-91.
24. Turner JA, Ersek M, Kemp C. Self-efficacy for managing pain is associated with disability, depression, and pain coping among retirement community residents with chronic pain. *The journal of pain : official journal of the American Pain Society* 2005;6(7):471-9. doi: 10.1016/j.jpain.2005.02.011
25. Connolly FR, Aitken LM, Tower M. An integrative review of self-efficacy and patient recovery post acute injury. *Journal of advanced nursing* 2014;70(4):714-28. doi: 10.1111/jan.12237
26. Middleton J, Tran Y, Craig A. Relationship between quality of life and self-efficacy in persons with spinal cord injuries. *Archives of physical medicine and rehabilitation* 2007;88(12):1643-8. doi: 10.1016/j.apmr.2007.09.001
27. Trost Z, Agtarap S, Scott W, et al. Perceived injustice after traumatic injury: Associations with pain, psychological distress, and quality of life outcomes 12 months after injury. *Rehabilitation psychology* 2015;60(3):213-21. doi: 10.1037/rep0000043
28. Trost Z, Monden KR, Buelow M, et al. Perceived Injustice Predicts Intention to Litigate: Findings from a Spinal Cord Injury Sample. *Psychological Injury and Law* 2016;9(1):31-40. doi: 10.1007/s12207-015-9245-z
29. Yakobov E, Scott W, Stanish W, et al. The role of perceived injustice in the prediction of pain and function after total knee arthroplasty. *Pain* 2014;155(10):2040-6. doi: 10.1016/j.pain.2014.07.007
30. Sullivan MJL, Thibault P, Simmonds MJ, et al. Pain, perceived injustice and the persistence of post-traumatic stress symptoms during the course of rehabilitation for whiplash injuries. *Pain* 2009;145(3):325-31. doi: 10.1016/j.pain.2009.06.031
31. Sullivan MJL, Scott W, Trost Z. Perceived injustice: A risk factor for problematic pain outcomes. *The Clinical journal of pain* 2012;8(6):484-88.



For peer review only - http://bmjopen.bmj.com/site/about/guidelines.xhtml

32. Sullivan MJL, Davidson N, Garfinkel B, et al. Perceived Injustice is Associated with Heightened Pain Behavior and Disability in Individuals with Whiplash Injuries. *Psychological Injury and Law* 2009;2(3-4):238-47. doi: 10.1007/s12207-009-9055-2
33. Monden KR, Trost Z, Scott W, et al. The unfairness of it all: Exploring the role of injustice appraisals in rehabilitation outcomes. *Rehabilitation psychology* 2016;61(1):44-53. doi: 10.1037/rep0000075
34. Gabbe BJ, Cameron PA, Williamson OD, et al. The relationship between compensable status and long-term patient outcomes following orthopaedic trauma. *Medical Journal of Australia* 2007;187(1):14-17.
35. McLean SA, Clauw DJ, Abelson JL, et al. The development of persistent pain and psychological morbidity after motor vehicle collision: integrating the potential role of stress response systems into a biopsychosocial model. *Psychosomatic medicine* 2005;67(5):783-90. doi: 10.1097/01.psy.0000181276.49204.bb
36. Sterling M, Hendrikz J, Kenardy J. Compensation claim lodgement and health outcome developmental trajectories following whiplash injury: a prospective study *Pain* 2010 150 22 - 8
37. Cameron PA, Gabbe BJ, McNeil JJ, et al. The trauma registry as a statewide quality improvement tool. *Journal of Trauma and Acute Care Surgery* 2005;59(6):1469-76.
38. Urquhart DM, Edwards ER, Graves SE, et al. Characterisation of orthopaedic trauma admitted to adult Level 1 Trauma Centres. *Injury-Int J Care Inj* 2006;37(2):120-27. doi: 10.1016/j.injury.2005.10.016
39. Williamson OD, Epi GDC, Gabbe BJ, et al. Predictors of moderate or severe pain 6 months after orthopaedic injury: a prospective cohort study. *Journal of orthopaedic trauma* 2009;23(2):139-44. doi: 10.1097/BOT.0b013e3181962e29
40. AAAM. Abbreviated Injury Scale 2005 update 2008. Barrington Illinois: Association for the Advancement of Automotive Medicine 2008.
41. Baker SP, O'Neill B, Haddon WJ, et al. The Injury Severity Score: a method for describing patients with multiple injuries and evaluating emergency care. *Journal of Trauma* 1974;14:187-96.
42. Cleeland CS, Ryan KM. Pain assessment: global use of the Brief Pain Inventory. *Annals of the Academy of Medicine, Singapore* 1994;23(2):129-38.
43. Gerbershagen HJ, Rothaug J, Kalkman CJ, et al. Determination of moderate-to-severe postoperative pain on the numeric rating scale: a cut-off point analysis applying four different methods. *British journal of anaesthesia* 2011;107(4):619-26. doi: 10.1093/bja/aer195
44. Kapstad H, Hanestad BR, Langeland N, et al. Cutpoints for mild, moderate and severe pain in patients with osteoarthritis of the hip or knee ready for joint replacement surgery. *BMC musculoskeletal disorders* 2008;9:55. doi: 10.1186/1471-2474-9-55
45. Wilson JT, Pettigrew LE, Teasdale GM. Structured interviews for the Glasgow Outcome Scale and the extended Glasgow Outcome Scale: guidelines for their use. *Journal of neurotrauma* 1998;15(8):573-85. doi: 10.1089/neu.1998.15.573 [published Online First: 1998/09/03]
46. McMillan T, Wilson L, Ponsford J, et al. The Glasgow Outcome Scale-40 years of application and refinement. *Nature Reviews Neurology* 2016;12(8):477-85. doi: 10.1038/nrneurol.2016.89

For peer review only



For peer review only - http://bmjopen.bmj.com/site/about/guidelines.xhtml

47. Ekegren CL, Hart MJ, Brown A, et al. Inter-rater agreement on assessment of outcome within a trauma registry. *Injury-Int J Care Inj* 2016;47(1):130-34. doi: 10.1016/j.injury.2015.08.002
48. Herdman M, Gudex C, Lloyd A, et al. Development and preliminary testing of the new five-level version of EQ-5D (EQ-5D-5L). *Quality of Life Research* 2011;20(10):1727-36. doi: 10.1007/s11136-011-9903-x
49. Dolan P. Modeling valuations for EuroQol health states. *Medical Care* 1997;35:1095-108.
50. Derrett S, Black J, Herbison GP. Outcome after injury-a systematic literature search of studies using the EQ-5D. *The Journal of trauma* 2009;67(4):883-90. doi: 10.1097/TA.0b013e3181ae6409 [published Online First: 2009/10/13]
51. Gabbe BJ, Simpson PM, Cameron PA, et al. Association between perception of fault for the crash and function, return to work and health status 1 year after road traffic injury: A registry-based cohort study. *BMJ Open* 2015;5(11):e009907. doi: 10.1136/bmjopen-2015009907
10.1136/bmjopen-2015-009907
52. Nicholas MK. The pain self-efficacy questionnaire: Taking pain into account. *European journal of pain* 2007;11(2):153-63.
53. Sullivan MJL, Adams H, Horan S, et al. The role of perceived injustice in the experience of chronic pain and disability: scale development and validation. *Journal of occupational rehabilitation* 2008;18(3):249-61. doi: 10.1007/s10926-008-9140-5
54. Miller RP, Kori S, Todd D. The Tampa Scale: a measure of kinesiophobia. *The Clinical journal of pain* 1991;7(1):51-52.
55. Shrout PE, Bolger N. Mediation in experimental and nonexperimental studies: new procedures and recommendations. *Psychological methods* 2002;7(4):422-45. [published Online First: 2003/01/18]
56. Sawilowsky SS. New effect size rules of thumb. *J Mod Appl Stat Methods* 2009;8(2):467–74.
57. Fritz MS, Mackinnon DP. Required sample size to detect the mediated effect. *Psychol Sci* 2007;18(3):233-9. doi: 10.1111/j.1467-9280.2007.01882.x
58. ABS. Qualifications and Work, Australia, 2015 (Cat. 4235.0). Canberra, Australia: Australian Bureau of Statistics, 2016.
59. ABS. Household Income and Wealth, Australia, 2013-14 (Cat 6523.0). Canberra, Australia: Australian Bureau of Statistics, 2015.
60. Murgatroyd DF, Cameron ID, Harris IA. Understanding the effect of compensation on recovery from severe motor vehicle crash injuries: a qualitative study. *Injury prevention : journal of the International Society for Child and Adolescent Injury Prevention* 2011;17(4):222-7. doi: 10.1136/ip.2010.029546
61. Thompson J, Berk M, O'Donnell M, et al. Attributions of responsibility and recovery within a no-fault insurance compensation system. *Rehabilitation psychology* 2014;59(3):247-55. doi: 10.1037/a0036543
62. Tait RC, Chibnall JT. Workers' Compensation Claimants with Low Back Pain: the Role of Dissatisfaction in the Transition to Disability. *Psychological Injury and Law* 2015;9(1):16-22. doi: 10.1007/s12207-015-9243-1
63. Scott W, Milioto M, Trost Z, et al. The relationship between perceived injustice and the working alliance: a cross-sectional study of patients with persistent pain attending multidisciplinary rehabilitation. *Disability and rehabilitation* 2016;38(24):2365-73. doi: 10.3109/09638288.2015.1129444



For peer review only - http://bmjopen.bmj.com/site/about/guidelines.xhtml

64. Sensky T. Chronic embitterment and organisational justice. *Psychotherapy and psychosomatics* 2010;79(2): 65-72. doi: doi: 10.1159/000270914
65. Asghari A, Nicholas MK. Pain self-efficacy beliefs and pain behaviour. A prospective study. *Pain* 2001;94(1):85-100.
66. Pierik JGJ, Ijzerman MJ, Gaakeer MI, et al. Incidence and prognostic factors of chronic pain after isolated musculoskeletal extremity injury. *European journal of pain* 2016;20(5):711-22. doi: 10.1002/ejp.796
67. Sullivan MJL, Adams H, Martel MO, et al. Catastrophizing and perceived injustice: risk factors for the transition to chronicity after whiplash injury. *Spine* 2011;36(25 Suppl):S244-9. doi: 10.1097/BRS.0b013e3182387fed
68. De Silva M, Maclachlan M, Devane D, et al. Psychosocial interventions for the prevention of disability following traumatic physical injury. *The Cochrane database of systematic reviews* 2009(4):CD006422. doi: 10.1002/14651858.CD006422.pub3
69. Wong EM, Chan SW, Chair SY. Effectiveness of an educational intervention on levels of pain, anxiety and self-efficacy for patients with musculoskeletal trauma. *Journal of advanced nursing* 2010;66(5):1120-31. doi: 10.1111/j.1365-2648.2010.05273.x [published Online First: 2010/03/27]
70. Yakobov E, Scott W, Thibault P, et al. Treatment-Related Reductions in Disability Are Associated with Reductions in Perceived Injustice Following Treatment of Whiplash Injury. *Psychological Injury and Law* 2016;9(1):41-47. doi: 10.1007/s12207-015-9248-9
71. Scott W, Trost Z, Bernier E, et al. Anger differentially mediates the relationship between perceived injustice and chronic pain outcomes. *Pain* 2013;154(9):1691-8. doi: 10.1016/j.pain.2013.05.015
72. Wade NG, Worthington EL. In Search of a Common Core: A Content Analysis of Interventions to Promote Forgiveness. *Psychotherapy: Theory, Research, Practice, Training* 2005;42(2):160-77. doi: 10.1037/0033-3204.42.2.160
73. Sullivan MJL, Yakobov E, Scott W, et al. Perceived Injustice and Adverse Recovery Outcomes. *Psychological Injury and Law* 2014;7(4):325-34. doi: 10.1007/s12207-014-9209-8
74. Scott W, McEvoy A, Garland R, et al. Sources of injustice among individuals with persistent pain following musculoskeletal injury. *Psychological Injury and Law* 2016;9(1):6-15. doi: 10.1007/s12207-015-9249-8

For peer review only



For peer review only - http://bmjopen.bmj.com/site/about/guidelines.xhtml

## FIGURE CAPTIONS

Figure 1. Study and analysis design.

Figure 2. Regression beta weights and ORs for the association between injury characteristics and psychological variables of pain catastrophizing, pain self-efficacy, kinesiophobia and injustice experience, adjusted for age, sex and injury severity *[Path a]*. Error bars (95% CI) that do not cross the central line indicate significant relationships. Tables of specific values can be found in Supplementary Materials.

Figure 3. Regression for association between baseline characteristics and functional recovery outcomes of pain interference (only for those with pain severity>0; N=370), EQ-5D, and GOS-E, adjusted for age, sex and injury severity. Error bars (95% CI) that do not cross the central line indicate significant relationships.

For peer review only

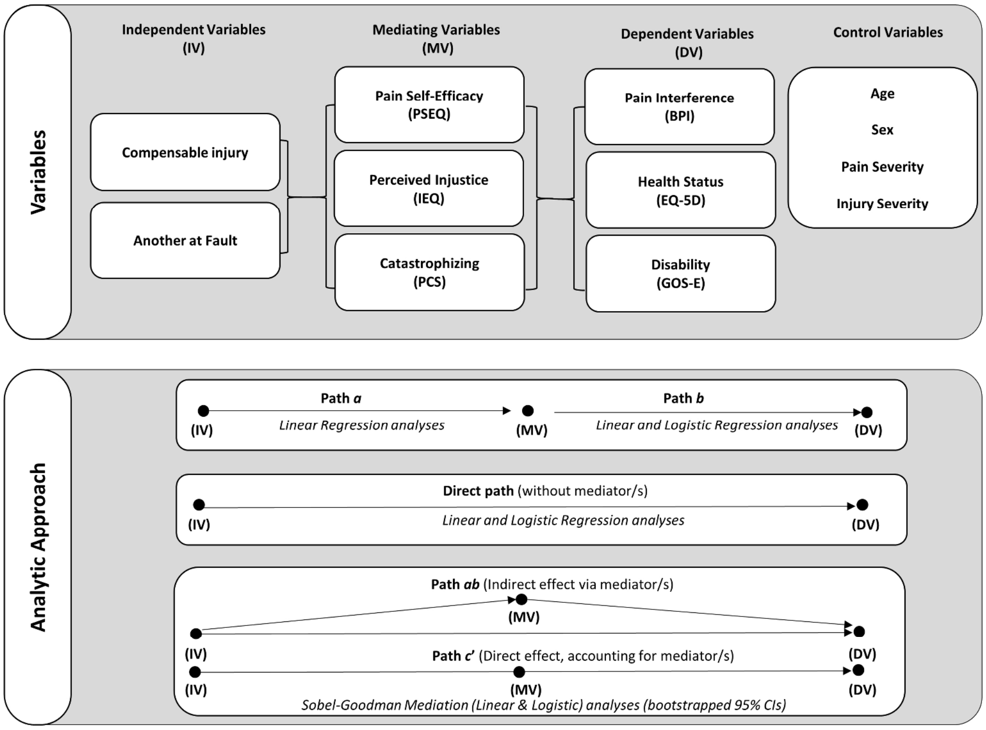


Figure 1. Study and analysis design.

254x190mm (300 x 300 DPI)

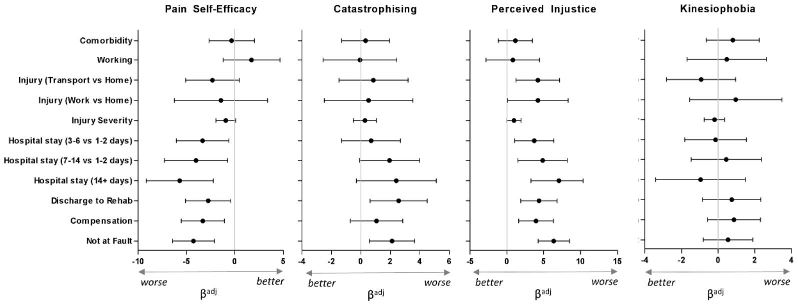


Figure 2. Regression beta weights and ORs for the association between injury characteristics and psychological variables of pain catastrophizing, pain self-efficacy, kinesiophobia and injustice experience, adjusted for age, sex and injury severity [Path a].
Notes: Error bars (95% CI) that do not cross the central line indicate significant relationships. Tables of specific values can be found in Supplementary Materials.

338x190mm (300 x 300 DPI)

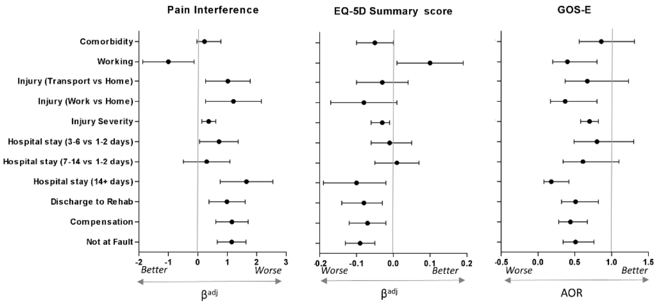


Figure 3. Regression for association between baseline characteristics and functional recovery outcomes of pain interference (only for those with pain severity>0; N=370), EQ-5D, and GOS-E, adjusted for age, sex and injury severity. Error bars (95% CI) that do not cross the central line indicate significant relationships.

338x190mm (300 x 300 DPI)

eview only

SUPPLEMENTARY

**Supplementary Table 1**

*Multiple regression for association between injury characteristics and pain interference (only for pain intensity>0; N=370), EQ-5D, and GOS-E recovery outcome.*

| | | Pain Interference | | | | EQ-5D Summary Score | | | | GOS-E Functional Outcome | | | |
|---|---|---|---|---|---|---|---|---|---|---|---|---|---|
| **Injury characteristics** | | **M** | **(sd)** | **β** | **(95% CI)** | **M** | **(sd)** | **β** | **(95% CI)** | **N** | **%** | **OR** | **(95% CI)** |
| **Comorbidities** | None | 2.91 | 2.42 | Ref | | 0.82 | 0.22 | Ref | | 137 | 50.6 | Ref | |
| | Present | 3.27 | 2.62 | 0.23 | -0.0.32,0.78 | 0.77 | 0.25 | -0.05 | -0.10,0 | 73 | 47.1 | 0.86 | 0.56,1.31 |
| **Work status at injury** | Not working | 3.97 | 2.87 | Ref | | 0.72 | 0.33 | Ref | | 37 | 68.5 | Ref | |
| | Working | 2.91 | 2.41 | **-1.00** | **-1.87,-0.13** | 0.82 | 0.21 | **0.10** | **0.01,0.19** | 173 | 46.5 | **0.40** | **0.20,0.80** |
| **Injury place** | At home | 2.48 | 2.39 | Ref | | 0.82 | 0.24 | Ref | | 44 | 57.9 | Ref | |
| | Traffic/Road | 3.60 | 2.61 | **1.02** | **0.26, 1.78** | 0.77 | 0.25 | -0.03 | -0.10,0.04 | 66 | 39.5 | 0.67 | 0.37,1.23 |
| | Workplace | 3.60 | 2.38 | **1.21** | **0.26, 2.16** | 0.74 | 0.25 | -0.08 | -0.17,0.01 | 14 | 31.1 | **0.37** | **0.17,0.80** |
| | Other | 2.43 | 2.24 | 0.15 | -0.62, 0.92 | 0.86 | 0.18 | 0.04 | -0.03,0.10 | 86 | 62.3 | 1.37 | 0.74,2.26 |
| **Injury severity** | AIS region count | | | **0.37** | **0.14, 0.61** | | | **-0.03** | **-0.06,-0.01** | | | **0.70** | **0.58,0.82** |
| **Hospital stay** | 1-2 days | 2.48 | 2.35 | Ref | | 0.83 | 0.24 | Ref | | 95 | 60.5 | Ref | |
| | 3-6 days | 3.26 | 2.46 | **0.72** | **0.06, 1.37** | 0.81 | 0.22 | -0.01 | -0.06,0.05 | 69 | 52.3 | 0.80 | 0.49,1.30 |
| | 7-13 days | 2.88 | 2.52 | 0.30 | -0.49, 1.09 | 0.82 | 0.19 | 0.01 | -0.05,0.07 | 35 | 43.8 | 0.61 | 0.34,1.10 |
| | ≥ 14 days | 4.34 | 2.49 | **1.65** | **0.76, 2.55** | 0.70 | 0.27 | **-0.10** | **-0.19,-0.02** | 10 | 17.9 | **0.18** | **0.08,0.42** |
| **Type of discharge** | Home | 2.65 | 2.29 | Ref | | 0.84 | 0.22 | Ref | | 168 | 55.6 | Ref | |
| | Rehabilitation | 3.91 | 2.72 | **0.99** | **0.38, 1.61** | 0.73 | 0.25 | **-0.08** | **-0.14,-0.03** | 42 | 33.9 | **0.51** | **0.32,0.82** |

For peer review only
Supplementary tables

| | | | | | | | | | | | | | |
|---|---|---|---|---|---|---|---|---|---|---|---|---|---|
| **Compensation status** | None | 2.53 | 2.32 | Ref | | 0.84 | 0.22 | Ref | | 155 | 58.7 | Ref | |
| | TAC/Worksafe | 3.82 | 2.57 | **1.16** | **0.61, 1.71** | 0.75 | 0.25 | **-0.07** | **-0.12,-0.02** | 55 | 34 | **0.44** | **0.28,0.67** |
| **Fault** | At fault | 2.42 | 2.14 | Ref | | 0.85 | 0.19 | Ref | | 124 | 57.9 | Ref | |
| | Not at fault | 3.63 | 2.68 | **1.15** | **0.66, 1.64** | 0.76 | 0.26 | **-0.09** | **-0.13,-0.05** | 83 | 39.9 | **0.51** | **0.34,0.76** |

*Notes*: all analysis adjusted for age, sex and injury severity, except for the injury severity analysis which only adjusted for age and sex.

For peer review only
Supplementary tables

Supplementary Table 2

*Multiple regression for association between injury characteristics, pain catastrophising, pain self-efficacy, kinesiophobia and injustice experience.*

| | | PCS | | | | PSEQ | | | |
|---|---|---|---|---|---|---|---|---|---|
| **Injury characteristics** | | **M** | **(sd)** | **β**** | **(95% CI)** | **M** | **(sd)** | **β**** | **(95% CI)** |
| **Comorbidities** | None | 9.54 | 11.23 | Ref | | 45.63 | 14.19 | Ref | |
| | Present | 9.30 | 10.6 | 0.32 | -1.31,1.94 | 45.02 | 14.68 | -0.34 | -2.68,2.01 |
| **Work status at injury** | Not working | 11.65 | 12.61 | Ref | | 40.99 | 15.12 | Ref | |
| | Working | 9.12 | 10.71 | -0.06 | -2.57,2.44 | 46.63 | 14.46 | 1.71 | -1.22,4.65 |
| **Injury place** | At home | 8.18 | 10.67 | Ref | | 47.41 | 12.01 | Ref | |
| | Traffic/Road | 11.38 | 11.81 | 0.85 | -1.49,3.20 | 42.7 | 15.27 | -2.32 | -5.09,0.46 |
| | Workplace | 11.13 | 11.3 | 0.52 | -2.48,3.53 | 43.11 | 15.41 | -1.44 | -6.26,3.38 |
| | Other | 7.18 | 9.49 | -1.27 | -3.55,1.00 | 48.43 | 13.37 | -0.10 | -2.97,2.76 |
| **Injury severity** | AIS region count | | | 0.28 | -0.51,1.06 | | | -0.94 | -1.96,0.08 |
| **Hospital stay** | 1-2 days | 7.91 | 10.00 | Ref | | 49.11 | 12.57 | Ref | |
| | 3-6 days | 9.77 | 11.3 | 0.69 | -1.31,2.68 | 43.81 | 14.99 | **-3.34** | **-6.05,-0.63** |
| | 7-13 days | 9.24 | 10.55 | 1.94 | -0.09,3.98 | 45.33 | 13.96 | **-4.02** | **-7.27,-0.77** |
| | ≥ 14 days | 13.25 | 12.76 | 2.40 | -0.31,5.11 | 38.89 | 15.5 | **-5.70** | **-9.16,-2.23** |
| **Type of discharge** | Home | 7.92 | 9.67 | Ref | | 47.41 | 13.61 | Ref | |
| | Rehabilitation | 13.08 | 12.97 | **2.56** | **0.63,4.50** | 40.71 | 15.01 | **-2.76** | **-5.10,-0.42** |
| **Compensation status** | None | 8.04 | 10.12 | Ref | | 47.91 | 13.29 | Ref | |
| | TAC/Worksafe | 11.67 | 11.93 | 1.06 | -0.72,2.84 | 41.43 | 15.12 | **-3.32** | **-5.55,-1.10** |

For peer review only

Supplementary tables

| | | | | | | | | | |
|---|---|---|---|---|---|---|---|---|---|
| **Fault** | At fault | 7.22 | 9.72 | Ref | | 48.94 | 12.07 | Ref | |
| | Not at fault | 11.67 | 11.80 | **2.11** | **0.57,3.65** | 41.87 | 15.63 | **-4.29** | **-6.45,-2.11** |

*Notes*: all analysis adjusted for age, sex and injury severity, except for the injury severity analysis which only adjusted for age and sex.

For peer review only
Supplementary tables

(Supplementary Table 2 continued)

| | | TSK | | | | IEQ | | | |
|---|---|---|---|---|---|---|---|---|---|
| **Injury characteristics** | | **M** | **(sd)** | **β**** | **(95% CI)** | **M** | **(sd)** | **β**** | **(95% CI)** |
| **Comorbidities** | None | 36.99 | 8.32 | Ref | | 15.99 | 13.6 | Ref | |
| | Present | 37.32 | 7.99 | 0.80 | -0.65,2.24 | 16.67 | 13.75 | 1.14 | -1.17,3.46 |
| **Work status at injury** | Not working | 37.81 | 9.33 | Ref | | 17.68 | 14.27 | Ref | |
| | Working | 37 | 8.01 | 0.47 | -1.70,2.64 | 16.02 | 13.55 | 0.80 | -2.83,4.42 |
| **Injury place** | At home | 36.97 | 7.86 | Ref | | 12.57 | 11.06 | Ref | |
| | Traffic/Road | 37.53 | 8.05 | -0.93 | -2.82,0.95 | 19.22 | 14.43 | **4.20** | **1.25,7.16** |
| | Workplace | 39.76 | 7.63 | 0.96 | -1.55,3.48 | 19.39 | 13.6 | **4.20** | **0.08,8.32** |
| | Other | 35.79 | 8.56 | -1.78 | -3.85,0.29 | 13.51 | 12.97 | 1.03 | -2.17,4.24 |
| **Injury severity** | AIS region count | | | -0.20 | -0.76,0.35 | | | 0.95 | -0.02,1.91 |
| **Hospital stay** | 1-2 days | 36.66 | 7.77 | Ref | | 12.54 | 12.57 | Ref | |
| | 3-6 days | 37.45 | 8.62 | -0.14 | -1.82,1.54 | 17.41 | 13.82 | **3.71** | **1.04,6.37** |
| | 7-13 days | 36.98 | 8.1 | 0.44 | -1.47,2.36 | 16.81 | 13.11 | **4.84** | **1.50,8.19** |
| | ≥ 14 days | 37.77 | 8.64 | -0.96 | -3.41,1.49 | 22.55 | 14.14 | **7.05** | **3.26,10.34** |
| **Type of discharge** | Home | 36.52 | 8.14 | Ref | | 14.09 | 12.87 | Ref | |
| | Rehabilitation | 38.53 | 8.18 | 0.74 | -0.85,2.33 | 21.31 | 14.12 | **4.35** | **1.88,6.81** |
| **Compensation status** | None | 36.3 | 8.05 | Ref | | 13.66 | 12.51 | Ref | |
| | TAC/Worksafe | 38.37 | 8.28 | 0.86 | -0.58,2.31 | 20.31 | 14.39 | **3.95** | **1.60,6.31** |

For peer review only

Supplementary tables

| | | | | | | | | | |
|---|---|---|---|---|---|---|---|---|---|
| **Fault** | At fault | 36.19 | 7.79 | Ref | | 11.94 | 11.1 | Ref | |
| | Not at fault | 38.03 | 8.54 | 0.54 | -0.81,1.89 | 20.5 | 14.62 | **6.35** | **4.23,8.48** |

*Notes*: all analysis adjusted for age, sex, pain intensity and injury severity.

For peer review only
Supplementary tables

Supplementary Table 3a

*Direct and indirect effects between predictors and pain interference, adjusting for age, sex, pain intensity and injury severity*

| | Indirect effects | | | | Direct effects |
|---|---|---|---|---|---|
| | B | (95% CI) | p-value | % of total effect mediated | p-value |
| **Mediation via Pain Self-Efficacy** | | | | | |
| Compensation status | 0.24 | 0.07,0.41 | 0.006 | 36.9% | 0.016 |
| Fault | 0.25 | 0.09,0.41 | 0.002 | 37.7% | 0.010 |
| **Mediation via Pain Catastrophising** | | | | | |
| Compensation status[a] | n/a | n/a | n/a | n/a | n/a |
| Fault | 0.17 | 0.03,0.32 | 0.017 | 26.0% | 0.001 |
| **Mediation via Perceived Injustice** | | | | | |
| Compensation status | 0.25 | 0.10,0.40 | 0.001 | 39.1% | 0.017 |
| Fault | 0.34 | 0.19,0.48 | <0.001 | 50.4% | 0.027 |

*Notes*, all analysis adjusted only for age, sex, pain intensity and injury severity. [a] Compensation status was not associated with catastrophising, so mediation not examined.

Supplementary Table 3b

*Combined mediation effects between predictors (fault attribution and compensation status) and pain interference*

| | **Fault attribution** | **Compensation** |
|---|---|---|
| Total indirect effect | | |
| β (95% CI) | 0.08 (0.04,0.12) | 0.07 (0.03,0.10) |
| p-value | <0.001 | <0.001 |
| % of mediated | | |
| Total effect | 59.3% | 48.7% |
| Pain self-efficacy | 23.9% | 25.1% |
| Perceived injustice | 19.3% | 23.6% |
| Pain Catastrophising | 16.2% | n/a |
| Direct effect (p-value) | | |
| β (95% CI) | 0.05 (0.00, 0.11) | 0.06 (0.00,0.12) |
| p-value | 0.055 | 0.051 |

*Notes*: all analysis adjusted only for age, sex, pain intensity and injury severity.

For peer review only
Supplementary tables

Supplementary Table 3c

*Direct and indirect effects between predictors and health status (EQ-5D), adjusting for age, sex and injury severity*

| | Indirect effects | | | | Direct effects [a] |
|---|---|---|---|---|---|
| | B | (95% CI) | p-value | % of total effect mediated | p-value |
| **Mediation via Pain Self-Efficacy** | | | | | |
| Compensation status | -0.016 | -0.029,-0.002 | 0.022 | 34.8% | 0.121 |
| Fault | -0.020 | -0.035,-0.004 | 0.012 | 33.5% | 0.026 |
| **Mediation via Pain Catastrophising** | | | | | |
| Compensation status[a] | n/a | n/a | n/a | n/a | n/a |
| Fault | -0.014 | -0.026,-0.002 | 0.026 | 24.1% | 0.011 |
| **Mediation via Perceived Injustice** | | | | | |
| Compensation status | -0.017 | -0.034,-0.008 | 0.008 | 37.0% | 0.151 |
| Fault | -0.026 | -0.042,-0.015 | <0.001 | 46.1% | 0.070 |

*Notes*, all analysis adjusted only for age, sex, pain intensity and injury severity. [a] Compensation status was not associated with catastrophising, so mediation not examined.

Supplementary Table 3d

*Combined mediation effects between predictors (fault attribution and compensation status) and EQ-5D*

| | **Fault attribution** | **Compensation** |
|---|---|---|
| Total indirect effect | | |
| β (95% CI) | -0.068 (-0.103,-0.033) | -0.048 (-0.079,-0.016) |
| p-value | <0.001 | 0.002 |
| % of mediated | | |
| Total effect | 54.0% | 50.1% |
| Pain self-efficacy | 21.7% | 26.0% |
| Perceived injustice | 16.9% | 24.1% |
| Pain Catastrophising | 15.5% | n/a |
| Direct effect (p-value) | | |
| β (95% CI) | -0.058 (-0.130,0.014) | -0.047 (-0.130,0.035) |
| p-value | 0.114 | 0.258 |

*Notes*, all analysis adjusted only for age, sex, pain intensity and injury severity.

For peer review only

Supplementary tables

Supplementary Table 3e

*Direct and indirect effects between predictors and functional outcome (GOS-E), adjusting for age, sex and injury severity*

| | Indirect effects | | | | Direct effects[a] |
|---|---|---|---|---|---|
| | B | (95% CI) | p-value | % of total effect mediated | p-value |
| **Mediation via Pain Self-Efficacy** | | | | | |
| Compensation status | -0.025 | -0.055,0.006 | 0.110 | 11.8% | 0.004 |
| Fault | -0.032 | -0.062,-0.001 | 0.041 | 19.4% | 0.032 |
| **Mediation via Pain Catastrophising** | | | | | |
| Fault | -0.026 | -0.054,0.002 | 0.065 | 16.4% | 0.023 |
| **Mediation via Perceived Injustice** | | | | | |
| Compensation status | -0.050 | -0.087,-0.013 | 0.009 | 23.5% | 0.006 |
| Fault | -0.084 | -0.125,-0.043 | <0.001 | 50.9% | 0.189 |

*Notes*, all analysis adjusted only for age, sex, pain intensity and injury severity.

Supplementary Table 3f

*Combined mediation effects for the relationship between GoS-E and compensation status/fault*

| | **Fault attribution** | **Compensation** |
|---|---|---|
| **Total indirect effect** | | |
| β (95% CI) | -0.089 (-0.133,-0.045) | -0.053 (-0.091,-0.016) |
| p-value | <0.001 | 0.006 |
| % of mediated | | |
| Total effect | 55.6% | 25.1% |
| Pain self-efficacy | 5.6% | 3.8% |
| Perceived injustice | 43.5% | 21.2% |
| Pain Catastrophising | 6.5% | n/a |
| **Direct effect (p-value)** | | |
| β (95% CI) | -0.071 (-0.192,0.051) | -0.159 (-0.278,-0.041) |
| p-value | 0.255 | 0.009 |

*Notes*, all analysis adjusted only for age, sex, pain intensity and injury severity.

For peer review only

Supplementary tables

**STROBE Statement—checklist of items that should be included in reports of observational studies**

**Manuscript title**: Associations between compensable injury, perceived fault and pain and disability one year after injury: A registry-based Australian cohort study

**Authors**: Giummarra et al.

| | Item No. | Recommendation | Page No. | Relevant text from manuscript |
|---|---|---|---|---|
| **Title and abstract** | 1 | (*a*) Indicate the study's design with a commonly used term in the title or the abstract | 2 | "Observational registry-based cohort study" |
| | | (*b*) Provide in the abstract an informative and balanced summary of what was done and what was found | 2 | |
| **Introduction** | | | | |
| Background/rationale | 2 | Explain the scientific background and rationale for the investigation being reported | 4-5 | |
| Objectives | 3 | State specific objectives, including any prespecified hypotheses | 5 | "We hypothesised that…" |
| **Methods** | | | | |
| Study design | 4 | Present key elements of study design early in the paper | 5+ | |
| Setting | 5 | Describe the setting, locations, and relevant dates, including periods of recruitment, exposure, follow-up, and data collection | 6-7 | |
| Participants | 6 | *Cohort study*—Give the eligibility criteria, and the sources and methods of selection of participants. Describe methods of follow-up<br>*Case-control study*—Give the eligibility criteria, and the sources and methods of case ascertainment and control selection. Give the rationale for the choice of cases and controls<br>*Cross-sectional study*—Give the eligibility criteria, and the sources and methods of selection of participants | 6-7 | |
| | | *Cohort study*—For matched studies, give matching criteria and number of exposed and unexposed<br>*Case-control study*—For matched studies, give matching criteria and the number of controls per case | n/a | |
| Variables | 7 | Clearly define all outcomes, exposures, predictors, potential confounders, and effect modifiers. Give diagnostic criteria, if applicable | 7-10 | |
| Data sources/ measurement | 8* | For each variable of interest, give sources of data and details of methods of assessment (measurement). Describe comparability of assessment methods if there is more than one group | 7-10 | |
| Bias | 9 | Describe any efforts to address potential sources of bias | 7 | "The present strategy to recruit from both VSTR |



For peer review only - http://bmjopen.bmj.com/site/about/guidelines.xhtml

| | | | | |
|---|---|---|---|---|
| | | | | and VOTOR …” |
| Study size | 10 | Explain how the study size was arrived at | 11 | “The sample (n = 433) was sufficiently powered…” |
| Quantitative variables | 11 | Explain how quantitative variables were handled in the analyses. If applicable, describe which groupings were chosen and why | 10-11 | Data analytic approach |
| Statistical methods | 12 | (*a*) Describe all statistical methods, including those used to control for confounding | 10-11 | |
| | | (*b*) Describe any methods used to examine subgroups and interactions | 10-11 | |
| | | (*c*) Explain how missing data were addressed | 10 | “participants with missing data …” |
| | | (*d*) *Cohort study*—If applicable, explain how loss to follow-up was addressed<br>*Case-control study*—If applicable, explain how matching of cases and controls was addressed<br>*Cross-sectional study*—If applicable, describe analytical methods taking account of sampling strategy | n/a | |
| | | (*e*) Describe any sensitivity analyses | n/a | |
| **Results** | | | | |
| Participants | 13* | (a) Report numbers of individuals at each stage of study—eg numbers potentially eligible, examined for eligibility, confirmed eligible, included in the study, completing follow-up, and analysed | 12 | |
| | | (b) Give reasons for non-participation at each stage | 12 | |
| | | (c) Consider use of a flow diagram | -- | |
| Descriptive data | 14* | (a) Give characteristics of study participants (eg demographic, clinical, social) and information on exposures and potential confounders | 12-13<br>Table 1 | |
| | | (b) Indicate number of participants with missing data for each variable of interest | Tables/footnotes | N included each analysis is reported in Tables 2 & 3, and Table 4 footnotes |
| | | (c) *Cohort study*—Summarise follow-up time (eg, average and total amount) | n/a | |
| Outcome data | 15* | *Cohort study*—Report numbers of outcome events or summary measures over time | n/a | |
| | | *Case-control study*—Report numbers in each exposure category, or summary measures of exposure | n/a | |
| | | *Cross-sectional study*—Report numbers of outcome events or summary measures | n/a | |
| Main results | 16 | (*a*) Give unadjusted estimates and, if applicable, confounder-adjusted estimates and their precision (eg, 95% confidence interval). Make clear which confounders were adjusted for and why they were included | 12-15<br>Table 3<br>Figure 1-2 | |
| | | (*b*) Report category boundaries when continuous variables were categorized | Indicated in tables where relevant | |
| | | (*c*) If relevant, consider translating estimates of relative risk into absolute risk for a meaningful time period | n/a | |
| Other analyses | 17 | Report other analyses done—eg analyses of subgroups and interactions, and sensitivity analyses | n/a | |



For peer review only - http://bmjopen.bmj.com/site/about/guidelines.xhtml

For peer review only

| | | | |
|---|---|---|---|
| **Discussion** | | | |
| Key results | 18 | Summarise key results with reference to study objectives | 22 |
| Limitations | 19 | Discuss limitations of the study, taking into account sources of potential bias or imprecision. Discuss both direction and magnitude of any potential bias | 20 |
| Interpretation | 20 | Give a cautious overall interpretation of results considering objectives, limitations, multiplicity of analyses, results from similar studies, and other relevant evidence | 25-26 |
| Generalisability | 21 | Discuss the generalisability (external validity) of the study results | Discussion (especially page 25) |
| **Other information** | | | |
| Funding | 22 | Give the source of funding and the role of the funders for the present study and, if applicable, for the original study on which the present article is based | 27 |

*Give information separately for cases and controls in case-control studies and, if applicable, for exposed and unexposed groups in cohort and cross-sectional studies.

**Note:** An Explanation and Elaboration article discusses each checklist item and gives methodological background and published examples of transparent reporting. The STROBE checklist is best used in conjunction with this article (freely available on the Web sites of PLoS Medicine at http://www.plosmedicine.org/, Annals of Internal Medicine at http://www.annals.org/, and Epidemiology at http://www.epidem.com/). Information on the STROBE Initiative is available at www.strobe-statement.org.

For peer review only



For peer review only - http://bmjopen.bmj.com/site/about/guidelines.xhtml
